# Benefits Associated with China’s Social Health Insurance Schemes: Trend Analysis and Associated Factors Since Health Reform

**DOI:** 10.3390/ijerph18115672

**Published:** 2021-05-25

**Authors:** Wanyue Dong, Anthony B. Zwi, Ruhai Bai, Chi Shen, Jianmin Gao

**Affiliations:** 1 School of Health Economics and Management, Nanjing University of Chinese Medicine, Nanjing 210023, China; wanyuedong@njucm.edu.cn; 2 School of Social Sciences, Faculty of Arts and Social Sciences, The University of New South Wales, Kensington 2052, Australia; 3 School of Public Affairs, Nanjing University of Science and Technology, Nanjing 210094, China; ruhaibai@hotmail.com; 4 School of Public Policy and Administration, Xi’an Jiaotong University, Xi’an 710049, China; shenchi@outlook.com

**Keywords:** social health insurance schemes, benefit trends, health reform, China

## Abstract

With the deepening of health insurance reform in China, the integration of social health insurance schemes was put on the agenda. This paper aims to illustrate the achievements and the gaps in integration by demonstrating the trends in benefits available from the three social health insurance schemes, as well as the influencing factors. Data were drawn from the three waves of the China Health and Nutrition Survey (2009, 2011, 2015) undertaken since health reforms commenced. χ^2^, Kruskal–Wallis test, and the Two-Part model were employed in the analysis. The overall reimbursement rate of the Urban Employee Basic Medical Insurance (UEBMI) is higher than that of Urban Resident Basic Medical Insurance (URBMI) or the New Rural Cooperative Medical Scheme (NRCMS) (*p* < 0.001), but the gap has narrowed since health reform began in 2009. Both the outpatient and inpatient reimbursement amounts have increased through the URBMI and NRCMS. Illness severity, higher institutional level, and inpatient service were associated with significant increases in the amount of reimbursement received across the three survey waves. The health reform improved benefits covered by the URBMI and NRCMS, but gaps with the UEBMI still exist. The government should consider more the release of health benefits and how to lead toward healthcare equity.

## 1. Introduction

Improving financial risk protection for all to achieve universal health coverage (UHC) is a key objective of the Sustainable Development Goals [1]. The Chinese government aims to achieve this goal by establishing a uniform social health insurance scheme developed from merging three social health insurance schemes: the Urban Employee Basic Medical Insurance (UEBMI), Urban Resident Basic Medical Insurance (URBMI), and the New Rural Cooperative Medical Scheme (NRCMS). Each targets a different population. Characteristics of the three social health insurance schemes in China are shown in Table 1.

These three social health insurance schemes (hereafter called “social health insurance schemes”) had been launched prior to 2009 and were each directed at different populations. The scope of benefits was limited, as reported by Yanfeng Ge as an important limitation of Chinese health reforms since the 1980s [2]. Thus, in April 2009, the Chinese central government announced new health system reforms which would involve public health services, medical care services, health insurance for financial protection, and an essential medicines policy to establish a universal healthcare system which would provide safe, efficient, and affordable basic healthcare services for all Chinese citizens by 2020 [3].

As a major component of consolidating the reforms, social health insurance was emphasized. In order to achieve the goal, the Chinese government invested over USD 800 billion from 2009 to 2015, particularly in subsidizing premiums for rural and urban residents who were not covered by the UEBMI to enable them to enroll in insurance schemes. As planned, substantial gains in social health insurance coverage have been achieved, and more than 95% of the Chinese population (about 1.336 billion people) were insured by the end of 2015 [4,5]. Many studies have evaluated the positive impact of health insurance. The expansion of social health insurance coverage had reduced out-of-pocket medical expenses from 56% in 2003 to 29% in 2017 [6]. It also has increased health service utilization and improved health outcomes [7,8]. However, more and more researchers have raised the issue of inequalities in healthcare given that the benefits of health insurance enjoyed by each subgroup of insurance enrollees are different [9]. As reported, the limited financial protection with reimbursement rates ranged from 44% to 68% [10]. Furthermore, the depth of coverage varies given the dissimilar financial protection of the people covered by different social health insurance schemes [11,12]. Consequently, those insured under different health insurance schemes are unequal in healthcare utilization, the choice of hospitals and healthcare cost [9].

The Chinese government realized that expanding the coverage of health insurance is the first step towards UHC. In response to the disparities among social health insurance schemes, policymakers aimed to merge these three packages into one over the next decade, although the name of this scheme has not yet been decided [13,14,15]. Global experiences indicate the importance of consolidating health insurance schemes for universal health coverage. In South Korea, three major types of health insurance were replaced by a national health insurance plan in 2000. While Japan has 3500 health insurance plans, the core elements of health insurance (i.e., co-payments and benefits) under these different health insurance plans are standardized [16]. By consolidating different health insurance plans into one system, inequities in financing and access to healthcare can be reduced [17]. Accordingly, integrating the fragmented social health insurance system to improve equity in access to health care has become a central issue for the Chinese government and researchers [4,18,19]. Thus, it is necessary to illustrate the different benefits among social health insurance schemes if integration is to be achieved.

As in South Korea which established a new administrative organization (the National Health Insurance Corporation) and Japan which provided premium subsidies from general revenues, in China a new institution, the “State Medical Insurance Administration”, was established in March 2018 [20]. Meanwhile, the government financing subsidy for the URBMI and NRCMS continuously increased from 80 CNY (100 CNY ≈ US$15.40, as at 31 December 2015) per person in 2008 to 380 CNY per person in 2015 to enhance the benefits associated with the insurance coverage [4,21,22,23,24]. However, how far away we are from integrating these social health insurance schemes has been little known so far. The breakdown of social health insurance has led to a gap in access to health care and financial protection among different groups of insured people [25,26,27]. However, limited research has analyzed the trend of health insurance benefits since the beginning of the reform. We hypothesize that the benefit gap among health insurance schemes should be gradually narrowed under the government’s efforts as mentioned above, after the execution of health reform. Besides, many studies demonstrated that the demand-side factors, including demographic, social, economic, and health status factors, were positively affecting enrolment in health insurance [28], but few researchers analyzed if these factors may affect the benefits of health insurance. Some studies compared reimbursement ratio, inpatient reimbursement amount on household registration or medical institution level [29,30], but it is still not yet clear how the general characteristics and health conditions of those who were insured and those receiving benefits affect health insurance benefits.

In this context, this research aims to demonstrate the trends in benefits among these key social health insurance schemes since health reform began in China, and to investigate the factors influencing these benefits. By exploring the extent to which health insurance consolidation has been achieved and determining what factors affect health insurance benefits, we hope to help researchers and policymakers better understand the achievements of the social health insurance system and future challenges in China.

## 2. Materials and Methods

Three waves (2009, 2011, 2015) of data were extracted from the China Health and Nutrition Survey Database (CHNS), which is an ongoing project held by the University of North Carolina and the Chinese Center for Disease Control and Prevention. CHNS used a multistage random-cluster sampling process to select samples from 15 provinces and municipal cities in China. The first wave of the CHNS was conducted in 1989, and subsequently in 1991, 1993, 1997, 2000, 2004, 2006, 2009, 2011 and 2015. In this study, after registration to access the data downloads, we mainly used information about health insurance, healthcare utilization, medical expenditure, family economic status, demographic characteristics and other information to conduct secondary data analysis. Detailed instructions about the database are available on the official website http://www.cpc.unc.edu/projects/china (accessed on 20 March 2021), the questionnaires are also presented in the Supplementary Material.

According to the research purpose, our sample only contains the latest data obtained in 2009 (the start year of health reform) and after. This study used repeated cross-sectional designs to evaluate health insurance benefits in different years. Only data from individuals above the age of 18 with social health insurance schemes and seeking health care from a formal medical provider in the previous four weeks before the interview in one of these three waves (2009, 2011, 2015) were included. A total of 2773 valid entries were utilized after processing the logical errors and removing incomplete or missing data.

In this study, we adopted two indicators to illustrate the benefits associated with social health insurance schemes: (i) reimbursement rate, namely the rate of reimbursement after the use of medical services, reflecting the breadth of benefit, and (ii) reimbursement amount, defined as the amount of reimbursement received for medical services to reflect the depth of benefit. We sought to remove (self-) selection bias by focusing on those who sought medical services in the prior four weeks instead of those who actively using the healthcare insurance benefits to which they are entitled [31]. The reimbursement amount was based on the costs of service and the percentage of cost sharing borne by the social health insurance schemes.

In the survey, the types of social health insurance schemes of adult members were reported by two questions “Do you have social health insurance?” [yes/no] and “What type of social health insurance do you have?” [UEBMI/URBMI/NRCMS]. The categories of medical institution level relied on the question “Where did you receive the services?” [clinic/township–community health centers/county hospitals/municipal hospitals and above]. Three types of severity of illness were available based on the question “How severe was the illness or injury?” [not severe/somewhat severe/quite severe]. The type of service was classified by the question “Was it an outpatient or inpatient visit?” [outpatient service/inpatient service]. In addition, the respondents were also asked “Do you buy any supplementary health insurance?” [yes/no], and “Was there a specific diagnosis of illness or injury by a doctor?” [yes/no].

Demographic and socioeconomic characteristics included age [18~44/45~59/60 and above], gender [male/female], marital status [married/others including single, divorced and widowed], educational level [primary school or below/middle school/technical school or above], employment status [yes/no], economic level [the poorest (from −2600 to 3700 CNY)/poorer (from 3713 to 7667 CNY)/medium (from 7670 to 13,978 CNY)/richer (from 14,000 to 25,183 CNY)/the richest (from 25,197 to 355,333 CNY)], and family size [2 or below/3–4/5 and above]. The economic level grouped by household income per capita was calculated by dividing household income by household size. Household income was conceptualized as the sum of all sources of income and revenue minus expenditures, including business, farming, fishing, gardening, livestock, non-retirement wages, retirement income, subsidies, and other income. Taking into account inflation, all costs in this study were made comparable, correcting for divided by the Consumer Price Index (CPI) obtained from State Statistics Bureau of China.

Descriptive statistics are the frequency and percentage of categorical variables, median and interquartile range (IQ range) of continuous variables. The categorical variables were tested by χ^2^ to assess whether the differences in social health insurance reimbursement rate were statistically significant. The 95% confidence intervals (CIs) for rates were calculated using a normal approximation. For comparison of the median reimbursement amount across different social health insurance schemes, the Kruskal–Wallis test was used.

Because of the large amount of zero medical expenses in the sample, which may undermine the assumption of random disturbances, this paper uses the two-part model (2 PM) proposed by Duan to analyze the social health insurance reimbursement data [32]. 2 PM assumes that two separate decision-making processes determine the benefit: Step (1) examines the probability of whether the benefit is obtained; Step (2) examines the determination of actual amount among the patient reimbursed by social health insurance schemes, with the log-transformed reimbursement amount as the dependent variable. The form of the 2 PM is expressed by the following equation:

(1) $$E\left(logy|x\right)=Pr\left(y>0|x\right)E\left(logy|y>0,x\right)$$

where Pr(y>0) is the likelihood of obtaining benefit from social health insurance schemes after seeking medical services, depending on a vector of observed characteristics x and specified using Logit regression in this study. OLS is used to estimate the reimbursement amount $E\left(logy|y>0,x\right)$. All analyses were performed using STATA statistical software version 14.0.

## 3. Results

### 3.1. Characteristics of Participants

The characteristics of the participants in each survey wave are presented in Table 2. Most participants were covered by the NRCMS (61.45% overall), followed by the UEBMI (22.32% overall). The proportion of the URBMI increased from 8.75% (2009) to 15.45% (2015). Slightly more than 40% of participants were male and elderly adults (≥60 years old), and the unemployment rose with year of survey. For all years, the marriage rate was stabilized at around 84%, and education was concentrated on primary and middle schools.

In terms of other insurance, more than 95% of participants did not have supplementary health insurance schemes. Over 86% of patients used outpatient services, and they usually chose clinics (41.94% overall) or municipal hospitals (23.40% overall), most health problems were somewhat severe or not severe (55.54% and 31.44% overall respectively), with a clear doctor’s diagnosis in 90.12% overall.

### 3.2. Reimbursement Rate of Different Social Health Insurance Schemes

**Figure 1:**
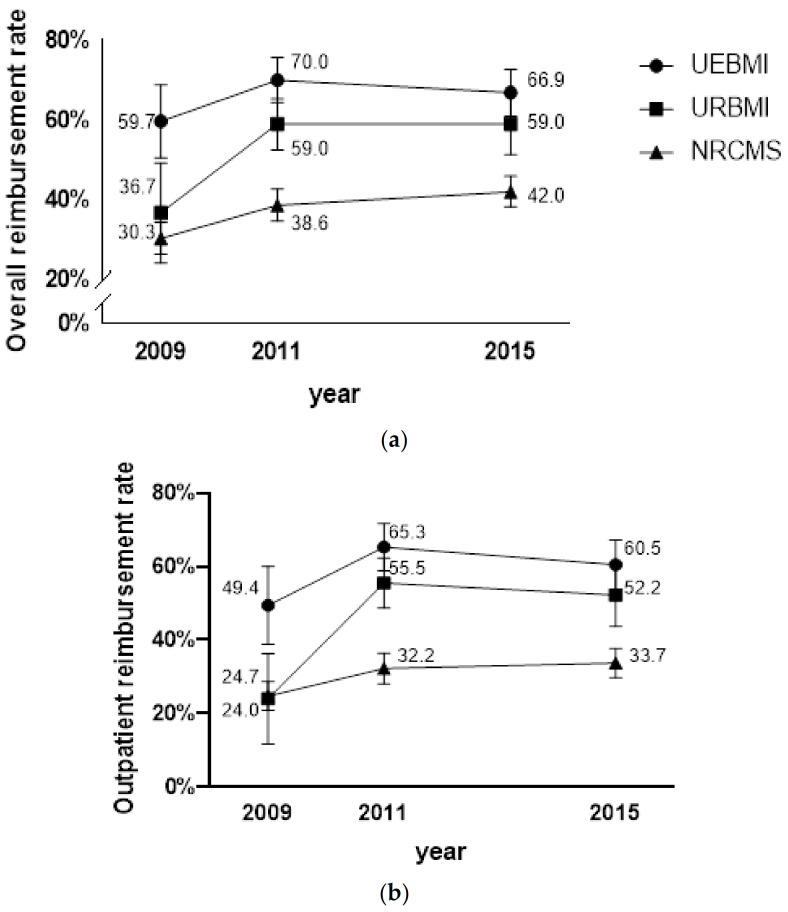

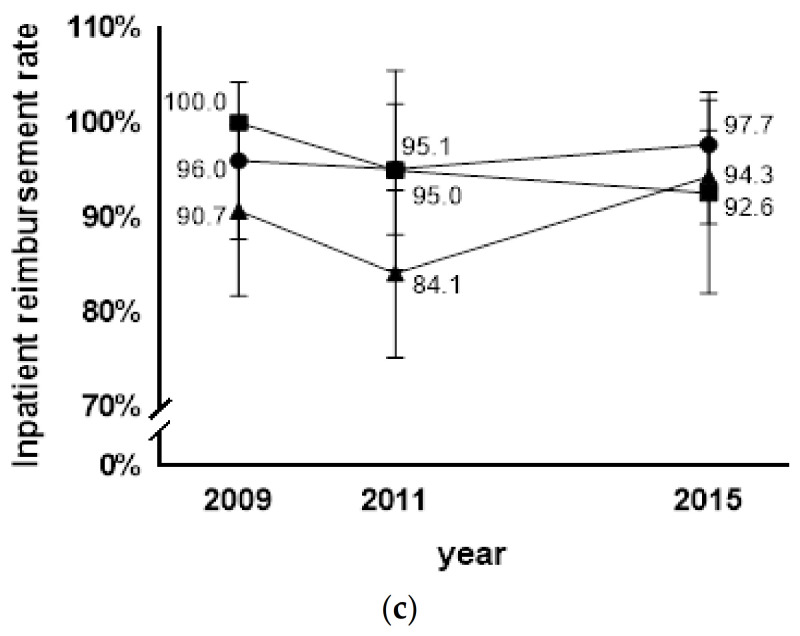
(**a**) Trend of overall reimbursement rate by year (with 95% confidence intervals) and social health insurance schemes in China; (**b**) Trend of outpatient reimbursement rate by year (with 95% confidence intervals) and social health insurance schemes in China; (**c**) Trend of inpatient reimbursement rate by year (with 95% confidence intervals) and social health insurance schemes in China.

A comparison of reimbursement rates through social health insurance schemes is presented in Figure 1. In 2009, the UEBMI had an overall rate of 59.7% (50.5–68.8%), higher than the URBMI of 36.7% (24.1–49.2%) and NRCMS of 30.3% (26.3–34.3%) (*p* < 0.001). Across the survey years, the overall reimbursement rates of both the URBMI and NRCMS increased, achieving 59.0% (51.3–66.7%) and 42.0% (38.2–45.9%) by 2015, respectively. Although the gap was narrowed among social health insurance schemes, the overall reimbursement rates were statistically different (all *p* < 0.001). In terms of outpatient reimbursement rates, the UEBMI had the highest rate of 60.5% (53.7–67.2%) in 2015, followed by the URBMI, which surpassed the NRCMS after 2009 to 55.5% (48.7–62.3%) in 2011 and 52.2% (43.7–60.8%) in 2015. Although the NRCMS has grown from 24.7% (20.8–28.7%) in 2009 to 33.7% (29.7–37.7%) in 2015, the gap was still large compared to the other two schemes. The inpatient reimbursement rates have reached over 90% in all social health insurance schemes, except for the NRCMS rate in 2011, which was 84.1% (75.2–92.9%).

### 3.3. Reimbursement Amount for the Different Social Health Insurance Schemes

Table 3, Table 4 and Table 5 show the reimbursement amount for the different social health insurance schemes. From 2009 to 2015, the overall amount decreased over time for the UEBMI, with a nadir in 2011. In contrast, the reimbursement amount of the NRCMS quadrupled until 2015. For the URBMI, the overall reimbursement amount fluctuated around 300–400 RMB. There was a statistically significant difference in the distribution of overall reimbursement amount among different social health insurance schemes in 2009 and 2011 (*p* < 0.05), but the difference became insignificant by 2015 (*p* = 0.393). For outpatient medical service, the reimbursement amount increased in the URBMI and NRCMS, but the differences among different social health insurance schemes were still statistical significantly in 2015 (*p* = 0.038). In contrast, the differences in the inpatient reimbursement amount among different social health insurance schemes became insignificant in 2015 (*p* = 0.131) with the further development of the URBMI and NRCMS.

### 3.4. Factors Associated with Benefits among Social Health Insurance Schemes

Table 6 shows the factors associated with reimbursement. Given the small numbers, analysis was not presented separately for outpatient or inpatient survey. The coefficients for insurance type were significantly negative in both reimbursement rate and the amount in 2009, but the coefficients became insignificant except for the reimbursement rate of the NRCMS in 2015, which indicates that the reform may have made social health insurance schemes more equitable. Higher severity and higher institutional level (above county hospitals) were associated with a significant increase in the amount reimbursed at the 1% level in three survey waves. With the deepening of health reforms, the reimbursement rate for seeking services in clinics was higher than in township–community health centers and county hospitals in 2015 (significantly at the 1% level). Inpatient service also increased the probability of getting reimbursement and the amount of reimbursement (significantly at the 1% level). Whether or not there was supplemental insurance did not affect the benefits of social health insurance schemes.

## 4. Discussion

Although China’s social health insurance system nominally covers everyone, due to the historical fragmentation of social health insurance schemes, finding out whether the program became more comprehensive and equitable after health reforms were commenced in 2009 and whether the gap to UHC has been reduced needs to be considered and evaluated [11]. Our findings suggest that the benefits of both the URBMI and NRCMS have increased, and the insurance gap has narrowed since health reform began in 2009. This study also shows that severity, medical institutional level, and type of service are associated with level of benefits.

Consistent with other studies, this study found that the benefit of health insurance schemes, especially the benefits of NRCMS, have increased [33,34]. However, it should be noted that our results also showed a decrease in the UEBMI overall reimbursement rate from 2011 to 2015. We attribute this to two reasons. Firstly, countries planning a significant expansion in health insurance coverage face a surge in health care [35], especially as outpatient services were included in the URBMI and NRCMS since 2011, the insured patients of the URBMI and NRCMS may place increased pressure on existing urban medical resources in the short term. It increased the time and transportation cost for the UEBMI insured, and affected their choice of whether to go to contracted hospitals and receive reimbursement. Secondly, different from the other two insurance types, part of the funds from both the employers and employees will be transferred to individual accounts for outpatient and emergency expenses in the UEBMI, which may serve as the basis of an accumulation of funds for use by the individual, to deal with the risks of catastrophic illnesses or the costs of long-term health care in the future. In particular, the increase of functional restrictions brought by ageing and less availability of informal family care will significantly affect the long-term care needs and services in the longevity of the ageing population. It may lead to the UEBMI insured suppressing their own needs to cope with higher long-term healthcare-related costs in the future [36].

A widely held view is that the fragmentation of the benefits among social health insurance schemes is an important barrier to equitable social development in China [37]. From the perspective of the gap among different health insurance schemes, the reimbursement rate of the UEBMI is higher than the URBMI and NRCMS [38]. This phenomenon has been widely reported in China [39,40], because the UEBMI is the health insurance scheme that grants its enrollees the most generous benefits [25]. This gap for urban and rural residents remains at about 10 percentage points throughout 2010 and 2016 in other studies [41].

In response to the gap, the Chinese government has endeavored to narrow the breadth and depth of benefits variation across social health insurance schemes and seems to be making progress in this direction [42]. The government has invested substantially in expanding the coverage of social health insurance schemes to unemployed urban and rural residents. Meanwhile, increasing subsidies were provided by the government for the URBMI and NRCMS. They are mainly dependent on government subsidies [43]. In addition, to enlarge the scope of insurance, outpatient services have been included in the URBMI and NRCMS since 2011 [44,45]. Moreover, the level of reimbursement also improved, and the reimbursement rates of the URBMI and NRCMS set a target of 50% and 75% for outpatient and inpatient service by 2015 [22,23]. Thus, the gaps in the distribution of reimbursement rates and amounts across different social health insurance schemes have continued to narrow, as shown in our study.

However, the above measures did not fundamentally make different health insurance schemes unify. Although the reimbursement policy has improved, the differences in health insurance schemes have caused imbalances. The reasons for this status quo are complex. In China, the benefit is determined by the level of funding in different health insurances, and the UEBMI, the scheme with the highest financial support, has relatively better service benefits and financial protection [46]. As mentioned earlier, the service benefits package of the URBMI and NRCMS initially focused on inpatient services. Although outpatient services were then included, a study found that outpatient reimbursement levels were deficient, ranging from 1% to 13% [47]. In addition, the fund coordinating units for different health insurance schemes are varied. The insurance funds of the UEBMI and URBMI are concentrated at the municipal level. In contrast, the funds of the NRCMS are concentrated at the county level, causing the problem of poor portability among different schemes [48].

The regression results of the two-part model showed that the coefficient of social health insurance gradually became insignificant, with the type of social health insurance scheme no longer being the determining factor affecting the amount of reimbursement in 2011 and 2015. Meanwhile, the coefficients of other factors largely operated in the desired direction. Higher severity, higher institutional level, and inpatient service were associated with significant increases in reimbursement amount in all years. Although the system is designed to obtain higher reimbursement in primary medical institutions, on the one hand, higher-level medical institutions are more likely to treat complex diseases, resulting in higher medical expenses leading to higher absolute reimbursement amounts [49]. On the other hand, since China does not have a general practitioner system that could serve as a gatekeeper in the health sector, Chinese patients typically prefer to seek medical care at higher levels of hospitals, and medical service prices charged by these hospitals are much higher, which means that the probability of exceeding the deductible level is greatly increased and the reimbursement amount is also increased [42]. In addition, in the initial design of social health insurance, one of the characteristics of the URBMI and NRCMS is to focus on catastrophic medical treatments by subsidizing hospitalization expenses, thereby increasing the possibility and amount of reimbursement for patients receiving inpatient services [17]. Some researchers believe that this compensation design had a negative effect, whether it led to insured who should be receiving outpatient treatment to over-utilize inpatient services, resulting in a waste of insurance funds [50]. In response to this problem, outpatient services were gradually added with the increase of funding, and the reimbursement standards of different health institutions levels were also implemented. Thus, with the deepening of health reform, the reimbursement rate for services sought by clinics was higher than that of township–community health centers and county hospitals in 2015, which may be partly attributed to the effect of triage of the patients. Whether there is supplementary insurance does not affect the benefits of the social health insurance. According to the design of China’s health insurance, no matter if it is critical illness insurance, medical assistance, or commercial health insurance, all exist as supplements to social health insurance, aiming to improve critical illness protection and respond to diverse health needs [51,52]. Our research showed that the social health insurance benefits would not be affected by supplementary insurance; those insured with supplementary insurance can get supplemental compensation after obtaining the benefits from social health insurances to meet their diversified financial protection needs.

The results of this study reflect the changes in the benefits among different social health insurance schemes in China’s long-running health reform, as well as the existing gaps to the unification of social health insurance schemes in China. Despite the achievements, progress in merging these schemes is still slow. The national government planned to merge the administrative authorities nationally in 2013, but the piloted integration reforms have failed to significantly reduce the difference in reimbursement rates and package coverage, presenting a high dissatisfaction rate with the ongoing integration reform among the staff members working in the units related to health insurance [53,54].

Our research reveals room for improvement in China towards the ultimate policy goal of integrating the social health insurance schemes into a unified system. First, compared with other countries, the financing of the NRCMS and the URBMI are heavily dependent on government subsidies. Therefore, the political and financial sustainability of the system cannot be ignored during the unification process [55]. Although the Chinese government has aimed at relieving the pressure of insurance funds caused by the rapid increase in medical expenses by establishing a tiered health service system, the effect is limited [41]. Therefore, the government needs to consider taking further public policy measures under a stable financing mechanism. Second, the design of a unified system requires careful consideration. In particular, the incomes of Chinese urban and rural residents are very different. The government needs to consider how to set an acceptable premium rate during the transition period to facilitate participants participating in the insurance according to their economic status, and to subsidize vulnerable groups so that everyone can enjoy the right to health [46]. Finally, improving the poor interconnection caused by the separation of health insurance management agencies is necessary. Unified management can promote the coordination of health insurance and reduce the financial pressure of operations.

In our study, the absolute amount reimbursed rather than the proportion was used to measure benefit, in order to eliminate concerns about the potential underutilization of healthcare services in disadvantaged groups [56]. In addition, using measures of reimbursement allows the elimination of confounding factors, such as the healthcare demands which may vary with income [11]. We therefore expect that the estimates of reimbursement rate and reimbursement amount are reliable.

Our study has several limitations. Accurate assessment of benefits could have been affected by the design of the scheme, including deductibles and reimbursement ceilings which could exhibit large variations, depending on the economic and financing level among regions and sectors [11,57]. Also, differences among groups (e.g., single, divorced and widowed respondents) were not considered due to the sample size limitations. Only married or “other” was considered. Another limitation is the possibility of limited representativeness. Our sample did not include individuals under the age of 18 and was restricted to those who sought medical care in the previous four weeks. Finally, other factors such as disease diagnosis prior to the survey or treatment method would be influential but have not been included due to limited data availability.

## 5. Conclusions

Through trend analysis of the benefits associated with China’s social health insurance schemes, this study found that implementing health reform effectively narrowed the difference in benefits among social health insurance schemes, including the reimbursement rate and reimbursement amount. The health reforms further improved healthcare equity, but the effect was relatively limited. It did not eliminate unequal overall reimbursement rates in 2015. Given the characteristics of different insurance schemes, we found that inpatient service, higher severity and higher institutional level were associated with significant reimbursement amounts.

This study suggests that the implementation of health insurance consolidation is necessary. Through measures such as establishing an institutional authority, optimizing the benefits package, and patient triage, China is attempting to develop its pathway to consolidating these different health insurance schemes. Whether China is aiming to replace these three types of health insurance with a new scheme or whether it seeks to integrate the existing schemes, the key concern will be to ensure that the core elements of health insurance are standardized after taking into account the vast differences in socioeconomic status and other key characteristics of those insured. In a further refinement of the schemes, the government should place emphasis on promoting healthcare equity as a key priority.

## Figures and Tables

**Table 1:** Characteristics of the three social health insurance schemes in China.

Features of Each Scheme	Urban Employee Basic Medical Insurance	Urban Resident Basic Medical Insurance	New Rural Cooperative Medical Scheme
	(UEBMI)	(URBMI)	(NRCMS)
Year established	1998	2007	2003
Target population	Urban employees	Urban unemployed, elderly, students, children	Rural residents
Risk-pooling unit	Municipal level	Municipal level	County level
Number of people insured by 2015 (millions)	288.93	376.89	670.00
Benefit package (in 2015)	Outpatient and inpatient care	Outpatient and inpatient care	Outpatient and inpatient care
Financing	Employer (6–8% of salary) Individual (2–3% of salary)	Government subsidy about 80% Individual about 20%	Government subsidy about 80% Individual about 20%

**Table 2:** Characteristics of participants [*n* (%)].

Variable	Setting	Total (*n* = 2773)	2009 (*n* = 686)	2011 (*n* = 1045)	2015 (*n* = 1042)
Insurance type	UEBMI	619 (22.32)	114 (16.61)	257 (24.59)	248 (23.80)
	URBMI	450 (16.23)	60 (8.75)	229 (21.92)	161 (15.45)
	NRCMS	1704 (61.45)	512 (74.64)	559 (53.49)	633 (60.75)
Gender	Male	1171 (42.23)	287 (41.84)	437 (41.82)	447 (42.90)
	Female	1602 (57.77)	399 (58.16)	608 (58.18)	595 (57.10)
Age (years)	18~44	516 (18.60)	161 (23.47)	217 (20.77)	138 (13.24)
	45~59	976 (35.20)	250 (36.44)	388 (37.12)	338 (32.44)
	≥60	1281 (46.20)	275 (40.09)	440 (42.11)	566 (54.32)
Marital status	Other	448 (16.16)	113 (16.47)	174 (16.65)	161 (15.45)
	Married	2325 (83.84)	573 (83.53)	871 (83.35)	881 (84.55)
Employment	No	1634 (58.93)	327 (47.67)	595 (56.94)	712 (68.33)
	Yes	1139 (41.07)	359 (52.33)	450 (43.06)	330 (31.67)
Education	≤Primary school	1304 (47.02)	391 (57.00)	453 (43.35)	460 (44.15)
	Middle school	1171 (42.23)	248 (36.15)	469 (44.88)	454 (43.57)
	≥Technical school	298 (10.75)	47 (6.85)	123 (11.77)	128 (12.28)
Family size	≤2	902 (32.52)	226 (32.94)	308 (29.47)	368 (35.32)
	3–4	1098 (39.60)	244 (35.57)	455 (43.54)	399 (38.29)
	≥5	773 (27.88)	216 (31.49)	282 (26.99)	275 (26.39)
Household income per capita	Poorest	546 (19.69)	177 (25.80)	196 (18.76)	173 (16.60)
	Poorer	550 (19.83)	192 (27.99)	208 (19.90)	150 (14.40)
	Medium	579 (20.88)	173 (25.22)	210 (20.10)	196 (18.81)
	Richer	551 (19.87)	95 (13.85)	236 (22.58)	220 (21.11)
	Richest	547 (19.73)	49 (7.14)	195 (18.66)	303 (29.08)
Supplementary insurance	No	2661 (95.96)	665 (96.94)	986 (94.35)	1010 (96.93)
	Yes	112 (4.04)	21 (3.06)	59 (5.65)	32 (3.07)
Severity of illness	Not severe	872 (31.44)	236 (34.40)	317 (30.34)	319 (30.61)
	Somewhat severe	1540 (55.54)	353 (51.46)	594 (56.84)	593 (56.91)
	Quite severe	361 (13.02)	97 (14.14)	134 (12.82)	130 (12.48)
Medical institution level	Clinic	1163 (41.94)	314 (45.77)	424 (40.58)	425 (40.79)
	Township/community health centers	565 (20.38)	123 (17.93)	228 (21.82)	214 (20.54)
	County hospitals	396 (14.28)	115 (16.76)	143 (13.68)	138 (13.24)
	Municipal hospitals and above	649 (23.40)	134 (19.54)	250 (23.92)	265 (25.43)
Service type	Outpatient	2408 (86.84)	608 (88.63)	915 (87.56)	885 (84.93)
	Inpatient	365 (13.16)	78 (11.37)	130 (12.44)	157 (15.07)
Diagnosed by doctor	No	274 (9.88)	45 (6.56)	75 (7.18)	154 (14.78)
	Yes	2499 (90.12)	641 (93.44)	970 (92.82)	888 (85.22)

Note: UEBMI: Urban Employee Basic Medical Insurance; URBMI: Urban Resident Basic Medical Insurance; NRCMS: New Rural Cooperative Medical Scheme.

**Table 3:** Overall reimbursement amount by year and social health insurance schemes in China (RMB).

Type	2009			2011			2015		
	Median	IQR		Median	IQR		Median	IQR	
		P25	P75		P25	P75		P25	P75
UEBMI	950	210	3750	340	100	1700	450	100	3230
URBMI	480	90	1100	225	90	600	300	78	1500
NRCMS	101	31.5	750	205	40	1350	400	64	1890
*p*-value	<0.001			0.007			0.393		

Note: UEBMI: Urban Employee Basic Medical Insurance; URBMI: Urban Resident Basic Medical Insurance; NRCMS: New Rural Cooperative Medical Scheme; IQR: interquartile range; P25: 25th Percentile; P75: 75th Percentile.

**Table 4:** Outpatient reimbursement amount by year and social health insurance schemes in China (RMB).

Type	2009			2011			2015		
	Median	IQR		Median	IQR		Median	IQR	
		P25	P75		P25	P75		P25	P75
UEBMI	280	127.5	1000	218	89	640	180	80	800
URBMI	90	36	480	180	80	425	187.5	60	350
NRCMS	70	22.5	153	91	30	335	130	40	500
*p*-value	<0.001			<0.001			0.038		

Note: UEBMI: Urban Employee Basic Medical Insurance; URBMI: Urban Resident Basic Medical Insurance; NRCMS: New Rural Cooperative Medical Scheme; IQR: interquartile range; P25: 25th Percentile; P75: 75th Percentile.

**Table 5:** Inpatient reimbursement amount by year and social health insurance schemes in China (RMB).

Type	2009			2011			2015		
	Median	IQR		Median	IQR		Median	IQR	
		P25	P75		P25	P75		P25	P75
UEBMI	3825	1995	5300	4000	1600	8000	4550	1700	8400
URBMI	1230	600	2400	3770	480	6800	5100	2800	8400
NRCMS	1320	420	2730	1700	850	3600	3200	1350	5700
*p*-value	<0.001			0.011			0.131		

Note: UEBMI: Urban Employee Basic Medical Insurance; URBMI: Urban Resident Basic Medical Insurance; NRCMS: New Rural Cooperative Medical Scheme; IQR: interquartile range; P25: 25th Percentile; P75: 75th Percentile.

**Table 6:** Regression results of factors associated with reimbursement rate and reimbursement amount in each survey wave using the two-part model.

Model Specification	Logit			Ordinary Least Squares		
	2009	2011	2015	2009	2011	2015
URBMI	−0.951 **	−0.219	−0.191	−0.653 *	−0.193	−0.0700
	(−2.25)	(−0.94)	(−0.77)	(−1.79)	(−1.08)	(−0.31)
NRCMS	−1.165 ***	−0.111	−0.741 ***	−0.711 **	−0.173	−0.0913
	(3.65)	(−0.47)	(−3.40)	(−2.55)	(−0.81)	(−0.42)
Somewhat severe	−0.0548	0.0285	−0.0891	0.722 ***	0.468 ***	0.351 *
	(−0.26)	(0.17)	(−0.53)	(3.30)	(2.93)	(1.95)
Quite severe	0.115	0.556 **	0.210	1.047 ***	1.166 ***	0.849 ***
	(0.35)	(2.00)	(0.75)	(3.63)	(5.17)	(3.31)
Township/community health centers	1.328 ***	1.221 ***	0.987 ***	0.381	−0.0789	−0.188
	(5.17)	(6.14)	(5.06)	(1.44)	(−0.42)	(−0.88)
County hospitals	1.067 ***	0.381	0.808 ***	1.571 ***	0.961 ***	0.946 ***
	(3.76)	(1.62)	(3.30)	(5.47)	(4.19)	(3.88)
Municipal hospitals and above	0.215	0.886 ***	0.322	1.613 ***	1.040 ***	0.984 ***
	(0.70)	(3.92)	(1.53)	(5.27)	(5.17)	(4.37)
Inpatient	3.354 ***	2.251 ***	2.957 ***	1.573 ***	1.926 ***	2.035 ***
	(6.61)	(6.97)	(7.92)	(6.96)	(10.78)	(10.64)
Supplementary insurance	−0.854	0.525	0.584	−0.926	−0.139	−0.625
	(−1.21)	(1.52)	(1.37)	(−1.24)	(−0.53)	(−1.63)
Diagnosed	0.529	0.674 **	0.378 *	0.0483	0.468	0.704 ***
	(1.22)	(2.12)	(1.78)	(0.09)	(1.36)	(2.60)
N	686	1045	1042	241	526	516

Note: URBMI: Urban Resident Basic Medical Insurance; NRCMS: New Rural Cooperative Medical Scheme; all specifications include controls for gender, age, marital status, employment, education, family size, and economic level; t statistics in parentheses; ***, ** and * denote statistical significance at 1, 5 and 10% level, respectively.

## Data Availability

“China Health and Nutrition Survey Database” at http://www.cpc.unc.edu/projects/china (accessed on 20 March 2021).

## Supplementary Materials

The following are available online at https://www.mdpi.com/article/10.3390/ijerph18115672/s1, Supplementary File S1: china Economic, population, nutrition, and health survey 2015 household questionnaire; Supplementary File S2: china economic, population, nutrition, and health survey 2015 individual questionnaire.

## Abbreviations

Universal health coverage (UHC); Urban Employee Basic Medical Insurance (UEBMI); Urban Resident Basic Medical Insurance (URBMI); New Rural Cooperative Medical Scheme (NRCMS); China Health and Nutrition Survey (CHNS); Consumer Price Index (CPI); interquartile range (IQR); confidence intervals (CIs); two-part model (2 PM).
