# Supplementary material for: Benefits Associated with China’s Social Health Insurance Schemes: Trend Analysis and Associated Factors Since Health Reform
Source: Int J Environ Res Public Health. 2021 May 25;18(11):5672. doi: 10.3390/ijerph18115672 (PMC8199469; doi:10.3390/ijerph18115672)
Supplement: Supplementary file 1 [file ijerph-18-05672-s001.zip › CHNS Questionnaire2015-Household.pdf]

CHINA ECONOMIC, POPULATION, NUTRITION, AND HEALTH SURVEY

2015HOUSEHOLD QUESTIONNAIRE

Province										
Province	11	Beijing	21	Liaoning	23	Heilongjiang	31	Shanghai	_ _	T1
	32	Jiangsu	37	Shandong	41	Henan	42	Hubei		
	43	Hunan	45	Guangxi	52	Guizhou	55	Chongqing		

Urban Site: 1 Rural Site: 2 _ T2

City: ____________________ County: _________________ _ T3

1	First city	1	First county	
2	Second city	2	Second county	
		3	Third county	
		4	Fourth county	

Neighborhood: _________________ Village (Town): ________________ _ _ T4

01	First [urban] neighborhood	01	County town neighborhood
02	Second [urban] neighborhood	02	First village
03	Third suburban village (neighborhood)	03	Second village
04	Fourth suburban village (neighborhood)	04	Third village
05	Fifth [urban] neighborhood	05	County town neighborhood
06	Sixth [urban] neighborhood	06	Fourth village
07	Seventh suburban village (neighborhood)	07	Fifth village
08	Eighth suburban village (neighborhood)	08	Sixth village
09	Ninth [urban] neighborhood	09	County town neighborhood
10	Tenth [urban] neighborhood	10	Seventh village
11	Eleventh suburban village (neighborhood)	11	Eighth village
12	Twelfth suburban village (neighborhood)	12	Ninth village

Household Address: ______District (Town) ______Street ______Apartment Number

Household Number: ____________ _ _ _ T5

Telephone Number: ____________

Interview Date: _ _ _ _Year _ _Month _ _Day _ _ _ _ _ _ _ _ T7

Completion Evaluation: 1 Good 2 OK 3 Poor _ CO

Interviewer Name: _______________ Number: _______________ _ _ T6c

Supervisor Name: ________________ Number: _______________ _ _ T6d

One Household questionnaire should be completed for each household, preferably by the head of the household. If the head of household cannot do this, another adult who is knowledgeable of the household's composition, basic information, and financial status should complete the questionnaire. The Household questionnaire includes the following sections:

	HOUSEHOLD ROSTER	
I	Household roster: Old members in old households	1
II	Household roster: New/returning members in old households and all members in new households	4
	DEMOGRAPHICS	
III	Background demographics of the household head (for new households only)	7
	WORK ACTIVITIES AND INCOME	
IV	Home gardening and income (for all households)	8
V	Household farming and income (for all households)	8
VI	Raising livestock/poultry and income (for all households)	8
VII	Household fishing and income (for all households)	10
VIII	Small handicraft and small commercial household business and income (for all households)	11
	OTHER INCOME	
IX	Income from other sources (for all households)	11
	DRINKING WATER, SANITATION, AND ASSETS	
X	Drinking water, environmental sanitation, and household assets (for all households)	13
XI	Household real estate (for all households)	14
XII	Household electrical appliances and other goods (for all households)	16
XIII	Household tools and equipment (for all households)	17
XIV	Household purchasing food or dishes online (for all households)	18

I. HOUSEHOLD ROSTER: OLD MEMBERS IN OLD HOUSEHOLDS (TABLE 1)
(for all households that participated in the study in 2011 and previous years)

* Check the information in Questions 1-5 in Table 1 for members who lived in the household in 2011 and previous years. If any information is incorrect, draw a line through it and record the correct information next to it (or above it). Please write the new information clearly.

* For most members who ever participated in the survey, two birth dates are provided with the CAPI programs: a Western date and a Lunar date. It is not necessary to check both dates. If the respondent knows one or both the dates are wrong, click on the button "Change Date of Birth" and record the right date and calendar.

If the correct date is known, but the calendar is not known, assume it is Western (record the date in the Western column).

Then ask Questions 7-12 for all members and record the answers in Table 1 (see Example 1/ Table 1 in training manual).

***After all questions in Table 1 are asked for all members who lived in the household in 2011 and previous years, go to Section II, ask Questions 1-13 for all new members, and record the answers in Table 2.**

1. Fill in the member's line number.

2. What is the member's name?

3. What is the member's sex?
   - 1 male
   - 2 female

4. What is the member's birth date? ____year ____month ____day

5. According to which calendar type (Western or Lunar)?

6. Did you change the birth date for this member?
   - 0 no
   - 1 yes

7. What is the member's relationship to the head of this household?
   - 00 head of household
   - 01 spouse
   - 02 father/mother
   - 03 son/daughter
   - 04 brother/sister
   - 05 grandson/granddaughter/ grandson-in-law/granddaughter-in-law
   - 06 father-in-law/mother-in-law
   - 07 son-in-law/daughter-in-law
   - 08 other relative (specify: __________)
   - 10 other non-relative (specify: _________)

8. Is this person still a household member?
   - 0 No
   - 1 yes (skip to Question 11)

9. When did this member move out of your house? ____year ____month
   * If "dead," record date of death, use code 7 for Question 10, then ask Questions 1-12 for next member.
   * If "moved out," record date of move, ask Question 10, then ask Questions 1-12 for next member.
   * If date is "unknown," record -99999.

10. Where does this member live now?
   - 1 same village/neighborhood
   - 2 same county
   - 3 same city
   - 4 same province
   - 5 other city, province (specify: __________)
   - 6 other country (specify: __________)
   - 7 Dead
   - 9 Unknown

   *If live in the same village/neighborhood, please recruit that household. Otherwise, stop here and ask Question 1-12 for next member.

11. Does this member still live in your household?
   - 1 yes (ask Q1-12 for next member)
   - 2 no, gone to school
   - 3 no, military service
   - 4 no, sought employment elsewhere
   - 5 no, gone abroad
   - 6 no, other (specify: __________)
   - 9 unknown

12. How long has this member been away from home? (months)
   * If "unknown," record -99.

Table 1. Household Roster: Old Members in Old Households

1<br>Line number	2<br>Name	3<br>Sex<br><br>1. M<br>2. F	4/5<br>Birth date<br>(year, month, day)<br>Western	Lunar	6<br>Was birth date changed?<br><br>0. No<br>1. Yes	7<br>Relationship to head of household<br><br>00. Head of household<br>01. Spouse<br>02. Father/mother<br>03. Son/daughter<br>04. Brother/sister<br>05. Grandson /granddaughter or in-law<br>06. Father/mother-in-law<br>07. Son/daughter-in-law<br>08. Other relative<br>10. Other non-relative	8<br>Still a household member?<br><br>0. No<br>1. Yes (skip to question 11)	9<br>When moved out?<br>(year, month)	10<br>Where lives now?<br><br>1.Same village/ neighborhood<br>2.Same county<br>3.Same city<br>4.Same province<br>5.Other city/province<br>6.Other country<br>7.Dead<br>9.Unknown<br><br>* go to next member	11<br>Still lives in your household?<br><br>1.Yes (ask Q1-12 for next member)<br>2.No, gone to school<br>3.No, military service<br>4.No, sought employment elsewhere<br>5.No, gone abroad<br>6.No, other<br>9.Unknown	12<br>How long gone?<br>(months)
**line**<br>_ _ _		**gender**<br>_	**west_dob**<br>_ _ _ _ _ _ _ _ _	**moon_dob**<br>_ _ _ _ _ _ _ _ _	**AA3c**<br>_	**A5**<br>_ _	**AA11**<br>_	**AA12**<br>_ _ _ _ _ _ _	**AA13**<br>_	**A5e**<br>_	**A5f**<br>_ _ _
_ _ _		_	_ _ _ _ _ _ _ _ _	_ _ _ _ _ _ _ _ _	_	_ _	_	_ _ _ _ _ _ _	_	_	_ _ _
_ _ _		_	_ _ _ _ _ _ _ _ _	_ _ _ _ _ _ _ _ _	_	_ _	_	_ _ _ _ _ _ _	_	_	_ _ _
_ _ _		_	_ _ _ _ _ _ _ _ _	_ _ _ _ _ _ _ _ _	_	_ _	_	_ _ _ _ _ _ _	_	_	_ _ _
_ _ _		_	_ _ _ _ _ _ _ _ _	_ _ _ _ _ _ _ _ _	_	_ _	_	_ _ _ _ _ _ _	_	_	_ _ _
_ _ _		_	_ _ _ _ _ _ _ _ _	_ _ _ _ _ _ _ _ _	_	_ _	_	_ _ _ _ _ _ _	_	_	_ _ _
_ _ _		_	_ _ _ _ _ _ _ _ _	_ _ _ _ _ _ _ _ _	_	_ _	_	_ _ _ _ _ _ _	_	_	_ _ _
_ _ _		_	_ _ _ _ _ _ _ _ _	_ _ _ _ _ _ _ _ _	_	_ _	_	_ _ _ _ _ _ _	_	_	_ _ _
_ _ _		_	_ _ _ _ _ _ _ _ _	_ _ _ _ _ _ _ _ _	_	_ _	_	_ _ _ _ _ _ _	_	_	_ _ _
_ _ _		_	_ _ _ _ _ _ _ _ _	_ _ _ _ _ _ _ _ _	_	_ _	_	_ _ _ _ _ _ _	_	_	_ _ _

II. HOUSEHOLD ROSTER: NEW MEMBERS IN OLD HOUSEHOLDS AND ALL MEMBERS IN NEW HOUSEHOLDS (TABLE 2)

(for all new members in old households and all members in newly formed households, replacement households and all households in replacement communities)

New Members in Old Households (see Example 1/Table 2):
* For new members in old households:
- ask Questions 1-13 and fill in Table 2
- if this member participated in the study as a member of a different household in the past, use code 1 for Question 9, and fill in the previous household ID number and line number in Questions 10-11
- assign a new line number (starting with 161) for Question 1

All Members in New Households:
* For all members in newly formed households (see Example 2), replacement households (see Example 3), and all households in replacement communities (see Example 4):
- ask Questions 1-13 and fill in Table 2
- use code 4 for Question 8
- assign a new household number starting with 161 to newly formed households or replacement households.
- assign a household number starting with 001 to households in replacement communities.
- assign a line number for each member of these households (starting with 001, not 161) for Question 1

1. Fill in the member’s line number.
2. What is the member’s name?
3. What is the member’s sex?
   1 male
   2 female
4. What is the member’s birth date? ____year ____month ____day
5. According to which calendar type (Western or Lunar)?
   * Record Western birth dates in the Western column. Record lunar birth dates in the lunar column.
   If calendar is unknown, assume Western. Record only one date for each member.
6. What is the member’s ethnicity (nationality)?
   01 Han
   06 Miao
   09 Buyi
   11 Man
   15 Tujia
   20 other (specify: __________)
   - 9 unknown
7. What is the member’s relationship to the head of this household?
   00 head of household
   01 spouse
   02 father/mother
   03 son/daughter
   04 brother/sister
   05 grandson/granddaughter/ grandson-in-law/granddaughter-in-law
   06 father-in-law/mother-in-law
   07 son-in-law/daughter-in-law
   08 other relative (specify: __________)
   10 other non-relative (specify: __________)
8. Under what circumstances did this member join this household?
   1 newborn
   2 marriage
   4 new household
   5 other (specify: __________)
9. Was this member a member of a household covered by this investigation previously (either the same household or a different household)?
   0 no (skip to Question 12)
   1 yes
   9 unknown (skip to Question 12)
10. What was the household ID number of the previous household?
   * Please look up the previous household ID and line number for this member, and record this information in Questions 10-11.
11. What was this member’s line number in the previous household?
12. Does this member still live in your household?
   1 yes (ask Q1-13 for the next member)
   2 no, gone to school
   3 no, military service
   4 no, sought employment elsewhere
   5 no, gone abroad
   6 no, other (specify: __________)
   9 unknown
13. How long has this member been away from home? (months)
   * If “unknown,” record -99.

Table 2. Household Roster: New/Returning Members in Old Households and All Members in New Households

1<br>Line number	2<br>Name	3<br>Sex<br><br>1. M<br>2. F	4/5<br>Birth date<br>(year, month, day)<br><br>Western	 <br><br><br>Lunar	6<br>Ethnicity<br>(nationality)<br><br>01. Han<br>06. Miao<br>09. Buyi<br>11. Man<br>15. Tujia<br>20. Other<br>-9. Unknown	7<br>Relationship to head of household<br><br>00. Head of household<br>01. Spouse<br>02. Father/mother<br>03. Son/daughter<br>04. Brother/sister<br>05. Grandson /granddaughter or in-law<br>06. Father/mother-in-law<br>07. Son/daughter-in-law<br>08. Other relative<br>10. Other non-relative	8<br>How joined this household?<br><br>1.Newborn<br>2.Marriage<br>4.New household<br>5.Other	9<br>In study before?<br><br>0. No (skip to Q 12)<br>1. Yes<br>9.Unknown (skip to Q 12)	10<br>Old household ID number<br><br>* Please look up the previous household ID and line number for this member, and record this information in Questions 10-11.	11<br>Old line number	12<br>Still lives in your household?<br><br>1.Yes (ask Q1-13 for next member)<br>2.No, gone to school<br>3.No, military service<br>4.No, sought employment elsewhere<br>5.No, gone abroad<br>6.No, other<br>9.Unknown	13<br>How long gone<br>(months)
**line**<br>_ _ _		**gender**<br>_	**west_dob**<br>_ _ _ _ _ _ _ _ _	**moon_dob**<br>_ _ _ _ _ _ _ _ _	**nationality**<br>_ _	**A5**<br>_ _	**AB5**<br>_	**AB6**<br>_	**AB7**<br>_ _ _ _ _ _ _ _ _ _	**AB8**<br>_ _ _	**A5e**<br>_	**A5f**<br>_ _ _
_ _ _		_	_ _ _ _ _ _ _ _ _	_ _ _ _ _ _ _ _ _	_ _	_ _	_	_	_ _ _ _ _ _ _ _ _ _	_ _ _	_	_ _ _
_ _ _		_	_ _ _ _ _ _ _ _ _	_ _ _ _ _ _ _ _ _	_ _	_ _	_	_	_ _ _ _ _ _ _ _ _ _	_ _ _	_	_ _ _
_ _ _		_	_ _ _ _ _ _ _ _ _	_ _ _ _ _ _ _ _ _	_ _	_ _	_	_	_ _ _ _ _ _ _ _ _ _	_ _ _	_	_ _ _
_ _ _		_	_ _ _ _ _ _ _ _ _	_ _ _ _ _ _ _ _ _	_ _	_ _	_	_	_ _ _ _ _ _ _ _ _ _	_ _ _	_	_ _ _
_ _ _		_	_ _ _ _ _ _ _ _ _	_ _ _ _ _ _ _ _ _	_ _	_ _	_	_	_ _ _ _ _ _ _ _ _ _	_ _ _	_	_ _ _
_ _ _		_	_ _ _ _ _ _ _ _ _	_ _ _ _ _ _ _ _ _	_ _	_ _	_	_	_ _ _ _ _ _ _ _ _ _	_ _ _	_	_ _ _
_ _ _		_	_ _ _ _ _ _ _ _ _	_ _ _ _ _ _ _ _ _	_ _	_ _	_	_	_ _ _ _ _ _ _ _ _ _	_ _ _	_	_ _ _
_ _ _		_	_ _ _ _ _ _ _ _ _	_ _ _ _ _ _ _ _ _	_ _	_ _	_	_	_ _ _ _ _ _ _ _ _ _	_ _ _	_	_ _ _
_ _ _		_	_ _ _ _ _ _ _ _ _	_ _ _ _ _ _ _ _ _	_ _	_ _	_	_	_ _ _ _ _ _ _ _ _ _	_ _ _	_	_ _ _

III. BACKGROUND DEMOGRAPHICS OF THE HOUSEHOLD HEAD
(for new households only)

1. Have you always lived here? _ A16
    0 no
    1 yes (skip to Question 3)

2. For how many years did you live elsewhere? _ _ A17

3. Are you a national minority? _ A19
    0 no
    1 yes

4. Where were you born? __________ province (region, city) _ _ A20

11	Beijing	21	Liaoning	31	Shanghai	41	Henan	51	Sichuan	61	Shaanxi
12	Tianjin	22	Jilin	32	Jiangsu	42	Hubei	52	Guizhou	62	Gansu
13	Hebei	23	Heilongjiang	33	Zhejiang	43	Hunan	53	Yunnan	63	Qinghai
14	Shanxi			34	Anhui	44	Guangdong	54	Tibet	64	Ningxia
15	Inner Mongolia			35	Fujian	45	Guangxi	55	Chongqing	65	Xinjiang
				36	Jiangxi	46	Hainan				
				37	Shandong						

5. Where is your "old home"? _________ province (region, city) _ _ A21

11	Beijing	21	Liaoning	31	Shanghai	41	Henan	51	Sichuan	61	Shaanxi
12	Tianjin	22	Jilin	32	Jiangsu	42	Hubei	52	Guizhou	62	Gansu
13	Hebei	23	Heilongjiang	33	Zhejiang	43	Hunan	53	Yunnan	63	Qinghai
14	Shanxi			34	Anhui	44	Guangdong	54	Tibet	64	Ningxia
15	Inner Mongolia			35	Fujian	45	Guangxi	55	Chongqing	65	Xinjiang
				36	Jiangxi	46	Hainan				
				37	Shandong						

6. Is your spouse a national minority? _ A26
    0 no
    1 yes
    9 no spouse or unknown (skip to next section)

7. Where was your spouse born? ______ province (region, city) _ _ A26a

11	Beijing	21	Liaoning	31	Shanghai	41	Henan	51	Sichuan	61	Shaanxi
12	Tianjin	22	Jilin	32	Jiangsu	42	Hubei	52	Guizhou	62	Gansu
13	Hebei	23	Heilongjiang	33	Zhejiang	43	Hunan	53	Yunnan	63	Qinghai
14	Shanxi			34	Anhui	44	Guangdong	54	Tibet	64	Ningxia
15	Inner Mongolia			35	Fujian	45	Guangxi	55	Chongqing	65	Xinjiang
				36	Jiangxi	46	Hainan				
				37	Shandong						

8. Where is your spouse's "old home"? ________ province (region, city) _ _ A27

11	Beijing	21	Liaoning	31	Shanghai	41	Henan	51	Sichuan	61	Shaanxi
12	Tianjin	22	Jilin	32	Jiangsu	42	Hubei	52	Guizhou	62	Gansu
13	Hebei	23	Heilongjiang	33	Zhejiang	43	Hunan	53	Yunnan	63	Qinghai
14	Shanxi			34	Anhui	44	Guangdong	54	Tibet	64	Ningxia
15	Inner Mongolia			35	Fujian	45	Guangxi	55	Chongqing	65	Xinjiang
				36	Jiangxi	46	Hainan				
				37	Shandong						

IV. HOME GARDENING AND INCOME (for all households)
***** We are asking about household income. If it is individual income, please record it into individual questionnaire.

1. Did your household have a vegetable garden or orchard in 2014? _ D1
   - 0 no (skip to the next section)
   - 1 yes
   - 9 unknown (skip to the next section)
2. In 2014, were any of the vegetables, fruits, or other produce from your home plot sold? _ D4
   - 0 no (skip to Question 4)
   - 1 yes
   - 9 unknown (skip to Question 4)
3. In 2014, how much money was received from the sale of the produce? (yuan) _ _ _ _ _ D5
   * If "unknown," record -9999.
4. In 2014, did your household consume any of the vegetables/fruits grown in your home plot? _ D6a
   - 0 No (skip to Question 6)
   - 1 Yes
   - 9 Unknown (skip to Question 6)
5. If the vegetables/fruits consumed by your household in 2014 had been sold, how much money do you think you would have received? (yuan) _ _ _ _ D6
   * If "unknown," record -999.
6. In 2014, how much money did you spend for seedlings, fertilizer, tools, insecticides, hired labor, etc. for this garden? (yuan) _ _ _ _ D7
   * This excludes farming tax and big machinery spending. If "unknown," record -999.

V. HOUSEHOLD FARMING AND INCOME (for all households)
***** We are asking about household income. If it is individual income, please record it into individual questionnaire.

1. Did your household engage in farming in 2014? _ E2b
   - 0 no(skip to the next section)
   - 1 yes
   - 9 unknown (skip to the next section)
2. How many mu of land did your household cultivate in 2014? (mu) _ _ _ E11d
   * If "unknown," record -99.
3. What was your household's total income from crops in 2014? (yuan) _ _ _ _ _ E14a
   * Crops include grains, tobacco, and greenhouse flowers. Income includes revenue from sales to the state and free market, and estimated value of crops on hand. If "unknown," record -9999.
4. In 2014, Did your household consume some of the crops grown? _ E16a1
   - 0 no(skip to Question 6)
   - 1 yes
   - 9 unknown (skip to Question 6)
5. If the crops consumed by your household in 2014 had been sold, how much money do you think you would have received? (yuan) _ _ _ _ _ E16a
   * If "unknown," record -9999.
6. In 2014, how much was spent for leasing land, for purchasing seedlings fertilizer, tools, insecticides, and hiring labor for these crops? (yuan) _ _ _ _ E12
   * If "unknown," record -999.

VI. RAISING LIVESTOCK/POULTRY AND INCOME (for all households)
***** We are asking about household income. If it is individual income, please record it into individual questionnaire.

1. Did your household raise livestock or poultry in 2014? _ F10a
   - 0 no(skip to next section)
   - 1 yes
   - 9 unknown (skip to next section)

*** Ask Questions 4-12 about each type of livestock/poultry raised by your household and record the answers in Table 3. If more than 4 types of livestock/poultry, record the 4 largest in scale (income).**

4. Did your household raise this kind of livestock or poultry in 2014?
   - 0 No (skip to the next type)
   - 1 Yes
5. In 2014, did your household sell any of this kind of livestock or poultry, or any products from them (eggs, milk, meat, wool, fertilizer, etc.)?
   - 0 no (skip to Question 7)
   - 1 yes
   - 2 did not raise this type of livestock (skip to the next type of livestock)
   - 9 unknown (skip to Question 7)
6. How much money did you receive? (yuan)
   * If "unknown," record -9999.
7. In 2014, did your household consume this kind of household-raised livestock or poultry, or products from them?
   - 0 no (skip to Question 9)
   - 1 yes
   - 9 unknown (skip to Question 9)
8. If the livestock or poultry or their products consumed by your household had been sold, how much money do you think you would have received? (yuan)
   * If "unknown," record -999.
9. In 2014, were any of the products of this kind of livestock or poultry, or livestock or poultry themselves, given away?
   - 0 no (skip to Questions 11)
   - 1 yes
   - 9 unknown (skip to Questions 11)
10. If the livestock, poultry, and the products given away had been sold, how much money do you think you would have received? (yuan)
    * If "unknown," record -999.
11. In 2014, how much money was spent for purchasing, feeding. and caring for this kind of livestock or poultry? (yuan)
    * If "unknown," record -999.
12. In 2014, was homemade animal feed given to this kind of livestock or poultry?
    - 0 no (ask Questions 4-13 for the next type)
    - 1 yes
    - 9 unknown (ask Questions 4-13 for the next type)
13. In 2014, how much money was saved by giving homemade feed to this kind of livestock or poultry? (yuan)
    * If "unknown," record -999.

Table 3. Raising Livestock/Poultry

2 Item numbe r	3 Livestock/ poultry type	4 Raised or not	5 Sold animals or products?	65 Amount received (yuan)	7 Consumed animals or products?	8 Value of animals/ products consumed (yuan)	9 Gave away animals or products?	10 Value of animals/ products given away (yuan)	11 Amount spent on care (yuan)	12 Fed homemade animal feed?	13 Amount saved (yuan)
**F11**		**F11a**	**F16**	**F17**	**F18**	**F19**	**F20**	**F21**	**F14**	**F15a**	**F15**
1	Pig, sheep	_	_	_ _ _ _ _	_	_ _ _ _	_	_ _ _ _	_ _ _ _	_	_ _ _ _
2	Chicken, duck, goose	_	_	_ _ _ _ _	_	_ _ _ _	_	_ _ _ _	_ _ _ _	_	_ _ _ _
3	Ox, horse, dog	_	_	_ _ _ _ _	_	_ _ _ _	_	_ _ _ _	_ _ _ _	_	_ _ _ _
4	Other	_	_	_ _ _ _ _	_	_ _ _ _	_	_ _ _ _	_ _ _ _	_	_ _ _ _

VII. HOUSEHOLD FISHING AND INCOME (for all households)

* We are asking about household income. If it is individual income, please record into individual questionnaire.

1. Did your household fish in 2014? _ G10a
    - 0 no (skip to the next section)
    - 1 Yes
    - 9 unknown (skip to the next section)

2. In 2014, how much money did your household receive from the fishing business? (yuan) _ _ _ _ _ G11
   * If "unknown," record -9999.

3. In 2014, did your household keep some fish for home consumption? _ G12
    - 0 no (skip to Question 5)
    - 1 Yes
    - 9 unknown (skip to Question 5)

4. If the fish kept for home consumption had been sold, how much money do you think you would have received? (yuan) _ _ _ _ G13
   * If "unknown," record -999.

5. In 2014, did your household give away fish? _ G14
    - 0 no (skip to Question 7)
    - 1 Yes
    - 9 unknown (skip to Question 7)

6. If the fish given away had been sold, how much money do you think you would have received? (yuan) _ _ _ _ G15
   * If "unknown," record -999.

7. In 2014, what were the total operating expenses of the household fishing business (gasoline, nets, lines, feed, fry, drugs, insurance, etc.)? (yuan) _ _ _ _ _ G16
   * If "unknown," record -9999.

VIII. SMALL HANDICRAFT AND SMALL COMMERCIAL HOUSEHOLD BUSINESS AND INCOME (for all households)

*** We are asking about household income. If it is individual income, please record into individual questionnaire.**

1. Did your household operate a small handicraft or small commercial business in 2014 (carpentry, shoe repair, housekeeping/child care service, tailoring, hairdressing, electrical appliance repair, restaurant, store, family child care, family hotel, family clinic, etc.)? _ H1
   - 0 no (skip to the next section)
   - 1 Yes
   - 9 unknown (skip to next section)

*** Ask Questions 4-6 about each business and record the answers in Table 4.**

*** If more than one business of the same type is reported, such as tailoring and hairdressing (both are services), add together the monthly revenues for these businesses and record the total for this type in Question 5 (H3). Then do the same for monthly expenses and record the total for this type in Question 6 (H4).**

Table 4. Small Household Businesses

2<br>Business number	3<br>Business type	4<br>Did your household operate this type of business in 2014?<br>0 no 1 yes<br>9 unknown<br>* If "no" or "unknown," skip down to next item.	5<br>What are the average monthly revenues of this business? (yuan)<br>* If "unknown," record -9999.	6<br>What are the average monthly expenses of this business, including salaries? (yuan)<br>* If "unknown," record -9999.
**H2**<br>1	Commerce	_	**H3**<br>_ _ _ _ _	**H4**<br>_ _ _ _ _
2	Service	_	_ _ _ _ _	_ _ _ _ _
3	Manufacturing	_	_ _ _ _ _	_ _ _ _ _
4	Peddler	_	_ _ _ _ _	_ _ _ _ _
5	Construction	_	_ _ _ _ _	_ _ _ _ _
6	Other (specify:___)	_	_ _ _ _ _	_ _ _ _ _

IX. INCOME FROM OTHER SOURCES (for all households)

*** Ask Questions 2-3 about each subsidy and record the answers in Table 5.**

Table 5. Subsidies

1<br>Subsidy	2<br>Did your household receive this subsidy in 2014?<br>0 no 1 yes 9 unknown<br>* If "no" or "unknown," skip down to next item.	3<br>How much money did your household receive? (yuan)<br>* If "unknown," record -99.
One-child cash subsidy	**_ I10**	**_ _ _ I10a**
Gas or fuel subsidy	**_ I15**	**_ _ _ I15a**
Coal subsidy	**_ I16**	**_ _ _ I16a**
Electricity subsidy	**_ I17**	**_ _ _ I17a**

4. In 2014, did your household (including all household members) receive any food gifts or discounted food from the work unit for spring festival or any other holidays? _ I20
   - 0 no (skip to Question 6)
   - 1 Yes
   - 9 unknown (skip to Question 6)

5. According to market prices, how much are these food gifts worth? (yuan) _ _ _ _ I21
   * If "unknown," record -999.

*** Ask Questions 7-8 about each income source and record the answers in Table 6.**

Table 6. Cash Income

6<br>Income Source	7<br>Did your household receive income from this source in 2014?<br>0 no 1 yes 9 unknown<br>* If "no" or "unknown," skip down to next item.	8<br>How much money did your household receive? (yuan)<br>*If "unknown," record -9999.
Rental of household assets, excluding land (houses, farm vehicles, farm equipment)	**_ J3a**	**_ _ _ _ _ _ J3**
Boarders or lodgers	**_ J4a**	**_ _ _ _ _ _ J4**
Poverty, disability, or welfare funds	**_ J6a**	**_ _ _ _ _ _ J6**
Money from children (non-household members)	**_ J7d**	**_ _ _ _ _ _ J7a**
Money from parents (non-household members)	**_ J7e**	**_ _ _ _ _ _ J7b**
Money from friends or other relatives	**_ J7f**	**_ _ _ _ _ _ J7c**
Cash income from other sources (excluding disaster relief)	**_ J8a**	**_ _ _ _ _ _ J8**

*** Ask Questions 10-11 about each type of gift and record the answers in Table 7.**

Table 7. Income In-Kind (Gifts)

9<br>Gift type	10<br>Did your household receive this type of gift in 2014?<br>0 no 1 yes 9 unknown<br>* If "no" or "unknown," skip down to next item.	11<br>What was the total value of these gifts? (yuan)<br>* If "unknown," record -999.
Gifts from children (non-household members)	**_ J9a**	**_ _ _ _ J9b**
Gifts from parents (non-household members)	**_ J9c**	**_ _ _ _ J9d**
Gifts from friends or other relatives	**_ J9e**	**_ _ _ _ J9f**
Money or gifts from local enterprise, such as bonuses (excluding salary income or bonus to a worker in the enterprise)	**_ J10a**	**_ _ _ _ J10b**

12. In total, what was your household income in 2014, including all sources of income such as salaries, bonus, and business income we talked above? (yuan) _ _ _ _ _ _B2e
    * If "unknown," record -99999.

X. DRINKING WATER, ENVIRONMENTAL SANITATION, AND HOUSEHOLD ASSETS (for all households)

1. How does your household obtain drinking water? _ L1

   * We are asking water you usually drink at home. We will ask about water you use to cook or wash food in questions 5 – 8.

   1 in-house tap water (skip to Question 3)
   2 in-yard tap water (skip to Question 3)
   3 in-yard well (skip to Question 3)
   4 Bottle water (skip to Question 3)
   5 other place (specify: __________)

2. How long does it take to walk to another place to get water? (minutes) _ _ _ L2

3. What is the source of this water? _ L3

   * If more than one source, record the most important one.

   1 ground water (>5 meters)
   2 open well (≤ 5 meters)
   3 creek, spring, river, lake
   4 ice/snow
   5 water plant
   6 Bottle water/ spring water or purified water
   7 other (specify: __________)
   9 unknown

4. Do you usually filter water before you drink? _ L1a

   0 No
   1 Yes

5. Do you use other source of water to cook or wash food? _ L1b

   0 No (skip to question 9)
   1 Yes

6. How does your household obtain water to cook or wash food? _ L1c

   1 in-house tap water (skip to Question 8)
   2 in-yard tap water (skip to Question 8)
   3 in-yard well (skip to Question 8)
   4 Bottle water (skip to Question 8)
   5 other place (specify: __________)

7. How long does it take to walk to another place to get water? (minutes) _ _ _ L2a

8. What is the source of this water? _ L3a

   * If more than one source, record the most important one.

   1 ground water (>5 meters)
   2 open well (≤ 5 meters)
   3 creek, spring, river, lake
   4 ice/snow
   5 water plant
   6 Bottle water
   7 other (specify: __________)
   9 unknown

9. Does your household pay for this drinking water or water for cooking? _ L4

   0 no
   1 yes

10. What kind of toilet facilities does your household have? _ L5
- 0 no bathroom
- 1 flush, in-house
- 2 no flush, in-house
- 3 flush, outside house, public restroom
- 4 no flush, outside house, public restroom
- 5 cement open pit
- 6 earth open pit
- 8 other (specify: _________)

11. Is there any excreta around the dwelling place? _ L6
* Record your own observation instead of asking the respondent.
- 1 no excreta
- 2 very little excreta
- 3 some excreta
- 4 much excreta

12. What kind of lighting does your household normally use? _ L7
- 1 electric
- 2 kerosene
- 3 oil
- 4 candle
- 5 other (specify: _________)

13. What kind of fuel does your household normally use for cooking? _ L8_1
* If there are 2 kinds, fill in L8_1 with the one most often used, and L8_2 with the second kind. If more than 2 kinds, record the 2 most often used. _ L8_2
If only one kind, fill in L8_1 only, and leave L8_2 blank.
- 1 coal
- 2 electricity
- 3 kerosene
- 4 liquified petroleum gas
- 5 natural gas
- 6 wood, sticks/straw, etc.
- 7 charcoal
- 8 other (specify: _________)

XI: HOUSEHOLD REAL ESTATE (for all households)

1. Do you own your house/apartment? _ L200
- 0 no (skip to Question 6)
- 1 yes

2. Did you buy your house/apartment? _ L201
- 0 no (skip to Question 8)
- 1 yes

3. Do you have a mortgage? _ L202
- 0 no (skip to Question 5)
- 1 yes

4. What is your monthly payment? _ _ _ _ _ L203
*** If unknown, record -999.**

5. What is your down payment (10,000 yuan)? _ _ _ _ L204
*** If unknown, record -999. If paid off at the first time, record the amount here; if built the house, record the total costs.**
*** Skip to question 8.**

6. Do you rent your house/apartment? _ L205
 0 no (skip to Question 8)
 1 yes

7. How much money per month do you pay for rent (yuan)? _ _ _ _ L10

8. What is the total usable area of your household's dwelling unit? (square meters) _ _ _ L16

9. Excluding the bathroom and toilet, how many rooms does your household have? _ _ L17

10. How much is this house/apartment worth? (10,000 yuan) _ _ _ _ L18
*** If does not know or is unwilling to estimate, record -999.**

11. Do you have other houses/apartments? _ L18a
 0 no (skip to the next section)
 1 yes

12. What is the total usable area of all other houses/apartment? (square meters) _ _ _ _ L18b

13. How much is all other houses/apartments worth? (10,000 yuan) _ _ _ _ L18c
*** If does not know or is unwilling to estimate, record -999.**

XII. HOUSEHOLD ELECTRICAL APPLIANCES AND OTHER GOODS (for all households)

*** Ask Questions 3-5 about each appliance and record the answers in Table 8.**

Table 8. Household Electrical Appliances

1<br>Item No.	2<br>Item name	3<br>Does your household own this appliance?<br>0 no 1 yes<br>* If "no," skip down to next item.	4<br>How many are owned?	5<br>How many were purchased in 2014?
L105a				
1	Color television	L105	L106	L107
2	Washing machine	_L110	L111	L112_
3	Refrigerator	_L115	L116_	L117_
4	Air conditioner	_L120	L121_	_L122
5	Sewing machine	_L125	_L126	L127_
6	Electric fan	_L130	_L131	L132_
7	Computer	L140e	L141e	L142e
8	Tablet computer	L140j	L141j	L142j
9	Camera	_L140	L141	L142
10	Microwave oven	_L140a	L141a	L142a
11	Electric rice cooker	_L140b	L141b	L142b
12	Pressure cooker	_L140c	L141c	L142c
13	Telephone	_L140f	L141f_	L142f_
14	Cell phone (non-smartphone)	_L140h	L141h_	L142h_
15	Smartphone	L140k_	L141k	_L142k
16	VCD or DVD	_L140g	L141g_	L142g_
17	Satellite Dish	_L140i	L141i_	L142i_
18	Exercise bikes/treadmills	_L140l	L141l	L142l
19	Massage chair	_L140m	L141m	L142m
20	Air purifier	_L140n	L141n	L142n

6. How many working TVs does your household have? _ L153

XIII. HOUSEHOLD TOOLS AND EQUIPMENT (for all households)

*** Ask Questions 2-4 about each means of transportation and record the answers in Table 9.**

Table 9. Means of Transportation

1<br>Transportation type	2<br>Does your household own this type of transportation?<br>0 no 1 yes 9 unknown<br>*If "no" or "unknown" skip down to next item	3<br>How many are owned	4<br>What is the value of all vehicles owned? (yuan)<br>*If "unknown", record -99999.
Tricycle	**_ L19**	**_ L20**	**_ _ _ _ L22**
Bicycle, including electric-bicycle	**_ L23**	**_ L24**	**_ _ _ _ L26**
Motorcycle, including motor tricycle	**_ L27**	**_ L28**	**_ _ _ _ _ _ L30**
Automobile	**_ L31**	**_ L32**	**_ _ _ _ _ _ L34**

*** Ask Questions 6-8 about each type of farm machinery and record the answers in Table 10.**

Table 10. Farm Machinery

5<br>Machinery type	6<br>Does your household own this type of machinery?<br>0 no 1 yes 9 unknown<br>* If "no" or "unknown," skip down to next item.	7<br>How many are owned?	8<br>What is the total value of all machines owned? (yuan)<br>* If "unknown," record -9999.
Tractor (large, medium, or small size)	**_ L37**	**_ L38**	**_ _ _ _ _ L40**
Garden tractor	**_ L41**	**_ L42**	**_ _ _ _ _ L44**
Irrigation equipment	**_ L49**	**_ L50**	**_ _ _ _ _ L52**
Power thresher	**_ L53**	**_ L54**	**_ _ _ _ _ L56**
Household water pump	**_ L57**	**_ L58**	**_ _ _ _ _ L60**
Other	**_ L57b**	**_ L58b**	**_ _ _ _ _ L60b**

*** Ask Questions 10-11 about each type of household commercial equipment and record the answers in Table 11. Ask Question 12 about all of the equipment reported in Table 11.**

Table 11. Household Commercial Equipment

9<br>Equipment type	10<br>Does your household own this type of equipment for use in business or an occupation to make money?<br>0 no 1 yes 9 unknown<br>* If "no" or "unknown," skip down to next item.	11<br>Was this equipment used for household business in 2014?<br>0 no 1 yes 9 unknown	12<br>What is the total value of all equipment owned? (yuan)<br>* If "unknown," record -9999.
Cooking equipment	**_ L74**	**_ L74a**	
Carpentry equipment	**_ L75**	**_ L75a**	
Haircut equipment	**_ L76**	**_ L76a**	
Sewing machine	**_ L77**	**_ L77a**	
Small machine shop tools or equipment	**_ L78**	**_ L78a**	
Other (specify: ______)	**_ L80**	**_ L80a**	**_ _ _ _ _ L81**

XIV. HOUSEHOLD PURCHSING FOOD OR DISHES ONLINE (for all households)

1. In the past month did your household purchase food from an online service? _ L300
   - 0 no (Finish Household Survey)
   - 1 Yes
   - 9 unknown (Finish Household Survey)

2. How many times did your household purchase food online in the past month? _ _ L301

3. About how much money did you spend the average month for food you purchase online (yuan)? _ _ _ _ L302

4. What are the major types of food you purchase?

(1)	Rice, wheat, and other cereal products	0 no	1 yes	_ L303
(2)	Meats, poultry, and eggs	0 no	1 yes	_ L304
(3)	Vegetables and fruits	0 no	1 yes	_ L305
(4)	Milk and dairy products	0 no	1 yes	_ L306
(5)	Beverages, cakes and cookies, candy, nuts or other snack foods	0 no	1 yes	_ L307
(6)	Condiments	0 no	1 yes	_ L308
(7)	Other processed foods	0 no	1 yes	_ L309

5. Why do you purchase food online?

(1)	Cheap	0 no	1 yes	_ L310
(2)	Convenient	0 no	1 yes	_ L311
(3)	Other (Specify____________)	0 no	1 yes	_ L312
