# Supplementary material for: Benefits Associated with China’s Social Health Insurance Schemes: Trend Analysis and Associated Factors Since Health Reform
Source: Int J Environ Res Public Health. 2021 May 25;18(11):5672. doi: 10.3390/ijerph18115672 (PMC8199469; doi:10.3390/ijerph18115672)
Supplement: Supplementary file 1 [file ijerph-18-05672-s001.zip › CHNS Questionnaire2015-Individua.pdf]

CHINA ECONOMIC, POPULATION, NUTRITION, AND HEALTH SURVEY
2015 INDIVIDUAL QUESTIONNAIRE
(For all participants age 0 and older)

Province	11	Beijing	21	Liaoning	23	Heilongjiang	31	Shanghai	32	Jiangsu	37	Shandong	_ _	T1
	41	Henan	42	Hubei	43	Hunan	45	Guangxi	52	Guizhou	55	Chongqing		

Urban Site: 1		Rural Site: 2		_	T2
City: ___________________		County: ________________		_	T3
1	First city	1	First county		
2	Second city	2	Second county		
		3	Third county		
		4	Fourth county		
Neighborhood: ________________		Village (Town): _______________		_ _	T4
01	First [urban] neighborhood	01	County town neighborhood		
02	Second [urban] neighborhood	02	First village		
03	Third suburban village (neighborhood)	03	Second village		
04	Fourth suburban village (neighborhood)	04	Third village		
05	Fifth [urban] neighborhood	05	County town neighborhood		
06	Sixth [urban] neighborhood	06	Fourth village		
07	Seventh suburban village (neighborhood)	07	Fifth village		
08	Eighth suburban village (neighborhood)	08	Sixth village		
09	Ninth [urban] neighborhood	09	County town neighborhood		
10	Tenth [urban] neighborhood	10	Seventh village		
11	Eleventh suburban village (neighborhood)	11	Eighth village		
12	Twelfth suburban village (neighborhood)	12	Ninth village		

Household Number: _______________		_ _ _	T5
Name: _______________	Line Number: __________	_ _ _	line
Telephone Number: _______________			
Name of Respondent: _______________	Line Number: __________	_ _ _	T6a
Interview Date: _ _ _ _Year _ _Month _ _Day		_ _ _ _ _ _ _ _	T7
Completion Evaluation: 1 Good	2 OK 3 Poor	_	CO
Interviewer Name: ______________	Number: ______________	_ _	T6c
Supervisor Name: _______________	Number: ______________	_ _	T6d

The Individual questionnaire should be completed for all participants. The Individual questionnaire includes the following sections:

	DEMOGRAPHICS	
I	BACKGROUND DEMOGRAPHICS (for all participants)	1
	WORK ACTIVITIES	
II	WORK STATUS (for participants age 16 and older)	3
III	PRIMARY OCCUPATION AND WAGES (for participants age 16 and older who work)	3
IV	SECONDARY OCCUPATION AND WAGES (for participants age 16 and older who work)	5
	INCOME	
V	HOME GARDENING (for participants age 6 and older)	6
VI	COLLECTIVE AND HOUSEHOLD FARMING (for participants age 6 and older)	6
VII	RAISING LIVESTOCK/POULTRY(for participants age 6 and older)	7
VIII	COLLECTIVE AND HOUSEHOLD FISHING (for participants age 6 and older)	7
IX	SMALL HANDICRAFT AND SMALL COMMERCIAL HOUSEHOLD BUSINESS (for participants age 6 and older)	8
X	OTHER SOURCES OF INCOME (for all participants)	9
	HOUSEHOLD CHORES AND CHILD CARE	
XI	TIME ALLOCATION FOR HOME ACTIVITIES (for participants age 6 and older)	9
XII	CARE OF CHILDREN AGE 6 AND YOUNGER (for participants age 6 and older)	9
XIII	CHILD CARE OUTSIDE THE HOME (for children age 6 and younger)	10
	SMOKING AND DRINKING	
XIV	SMOKING (for participants age 12 and older)	10
XV	ALCOHOL CONSUMPTION (for participants age 15 and older)	11
	PHYSICAL ACTIVITIES	
XVI	PHYSICAL ACTIVITIES (for children under age 6)	11
XVII	PHYSICAL ACTIVITIES (for participants age 6 and older who are in school)	12
XVIII	PHYSICAL ACTIVITIES (for participants age 6 and older who are not in school)	14
XIX	ACCESS TO THE INTERNET (for participants age 6 and older)	16
XX	BODY SHAPE AND MASS MEDIA (for participants age 6 to 17)	17
XXI	ACTIVITIES OF DAILY LIVING (for participants age 55 and older)	19
XXII	MEMORY TEST (for participants age 55 and older)	20
	HEALTH SERVICES AND DISEASE HISTORY	
XXIII	MEDICAL INSURANCE (for all participants)	21
XXIV	USE OF HEALTH CARE AND MEDICAL SERVICES (for all participants)	22
XXV	DISEASE HISTORY (for participants age 6 and older)	24
XXVI	PERCEIVED STRESS (for participants age 18 and older)	27
	DIET AND ACTIVITY KNOWLEDGE	
XXVII	DIET AND ACTIVITY KNOWLEDGE (for participants age 12 and older)	27
XXVIII	FRQUENCY OF CONSUMING SNACKS AND PACKAGED FOODS (for participants age 6 and older)	30
	EVER-MARRIED WOMEN	
XXIX	MARRIAGE HISTORY (for all women under age 52 who are married, widowed, or divorced)	31
XXX	INTER-GENERATIONAL LINKAGES TO PARENTS (for all women under age 52 who are married, widowed, or divorced)	32
XXXI	SIBLINGS/RELATIVES (for all women under age 52 who are married, widowed, or divorced)	32
XXXII	PREGNANCY HISTORY (for all women under age 52 who are married, widowed, or divorced)	33
XXXIII	BIRTH HISTORY (for all women under age 52 who are married, widowed, or divorced, and who have given birth to a child)	34
	EATING DISORDER	
XXXIV	EATING DISORDER (for women age 12-49 years)	35
	PHYSICAL MEASUREMENTS	
XXXV	FIRST MENSTRUATION (for girls age 8 and older)	36
XXXVI	Boy Maturation (for all boys 10 years old and older)	36

I. BACKGROUND DEMOGRAPHICS (for all participants)

1. Date of birth: ____year ____month ____day _ _ _ _ _ _ _ _AA3

   * Record western calendar, if possible, and use the same date of birth in household questionnaire.

2. According to which calendar type? _ AA4
   - 1 western calendar
   - 2 lunar calendar

3. Age (years): ________ _ _ _ age
   * Record 018 if 18.00-18.99 years, 019 if 19.00-19.99 years, etc.

4. Sex: _______ _ gender
   - 1 male
   - 2 female

5. What is the current name of province where were you born? _ _ A20a

11	Beijing	21	Liaoning	31	Shanghai	41	Henan	51	Sichuan	61	Shaanxi
12	Tianjin	22	Jilin	32	Jiangsu	42	Hubei	52	Guizhou	62	Gansu
13	Hebei	23	Heilongjiang	33	Zhejiang	43	Hunan	53	Yunnan	63	Qinghai
14	Shanxi			34	Anhui	44	Guangdong	54	Tibet	64	Ningxia
15	Inner Mongolia			35	Fujian	45	Guangxi	55	Chongqing	65	Xinjiang
				36	Jiangxi	46	Hainan				
				37	Shandong						

6. Is the settlement where you were born a city, a suburban, a county capital city, or a village? _ A20b
   - 1 A city
   - 2 A suburban
   - 3 A county capital city
   - 4 A village

7. Does your father live in this household? _ A5a
   - 0 no (skip to Question 10)
   - 1 Yes

8. What is the relationship between you and your father? _ A5a1
   - 1 biological father
   - 2 stepfather
   - 3 adopted father

9. What is your father's name? __________ _ _ _ A5b
   * Record the father's line number.

10. Does your mother live in this household? _ A5c
    - 0 no (skip to Question 13)
    - 1 Yes

11. What is the relationship between you and your mother? _ A5c1
    - 1 biological mother
    - 2 stepmother
    - 3 adopted mother

12. What is your mother's name? __________ _ _ _ A5d
    * Record the mother's line number.

13. To which type of household registration do you belong? _ A8b1
    - 1 urban
    - 2 rural

* If age 6 or older, ask Questions 14-19. Otherwise, skip to Section XIII.

14. How many years of formal education have you completed in a regular school? _ _ A11

00	no school completed (skip to Q16)	26	3 years upper middle school
11	1 year primary school (skip to Q16)	27	1 year technical school
12	2 years primary school (skip to Q16)	28	2 years technical school
13	3 years primary school (skip to Q16)	29	3 years technical school
14	4 years primary school (skip to Q16)	31	1 year college/university
15	5 years primary school	32	2 years college/university
16	6 years primary school	33	3 years college/university
21	1 year lower middle school	34	4 years college/university
22	2 years lower middle school	35	5 years college/university
23	3 years lower middle school	36	6 years college/university or more
24	1 year upper middle school	- 9	unknown
25	2 years upper middle school		

15. What is the highest level of education you have attained? _ A12
    - 1 graduated from primary school
    - 2 lower middle school degree
    - 3 upper middle school degree
    - 4 technical or vocational degree
    - 5 university or college degree
    - 6 master's degree or higher
    - 9 unknown

16. Are you currently in school? _ A13
    - 0 no (skip to Q20 if age≥ 18; otherwise skip to the next section)
    - 1 yes

17. During the school semester do you live away from home in or near school? _ A13a
    - 0 no (skip to Q20 if age≥ 18; otherwise skip to the next section)
    - 1 yes

18. Do you go home for each weekend? _ A13b
    - 0 no
    - 1 yes

19. How old were you when you first lived away from home in or near school? _ _ A13c

*** If age 18 and older, ask questions 20-21. Otherwise, skip to the next section.**

20. What is your marital status? _ A8
    - 1 never married (skip to the next section)
    - 2 married
    - 3 divorced (skip to the next section)
    - 4 widowed (skip to the next section)
    - 5 separated (skip to the next section)
    - 9 unknown (skip to the next section)

21. What is your spouse's name? __________ _ _ _ A8b
    * Record the spouse's line number.

II. WORK STATUS (for participants age 16 and older)

1. Are you presently working? _ B2
   * If retired but rehired, record 1.
   - 0 No
   - 1 yes (skip to Question 3)

2. Why are you not working? _ B2a
   - 1 seeking work (skip to Section V)
   - 2 doing housework (skip to Section V)
   - 3 disabled (skip to Section V)
   - 4 student (skip to Section V)
   - 5 retired (skip to Question 4)
   - 6 Too young to work (skip to Section V)
   - 7 other (specify: __________) (skip to Section V)
   - 9 unknown (skip to Section V)

3. Are you retired, but rehired? _ B2b
   - 0 no (skip to Question 6)
   - 1 yes

4. When did you retire? ____year ____month _ _ _ _ _ _ B2c
   * Record western calendar, if possible. If year and month are unknown, record -99999.

5. On the average, what was your monthly retirement wage/salary in 2014, including subsidies and bonuses? (yuan) _ _ _ _ B2d
   * If unknown, record -999.

   *** If retired, but rehired, ask Question 6. Otherwise, skip to Section V**

6. Did you change your job after 2011? _ B3b
   - 0 no
   - 1 yes

III. PRIMARY OCCUPATION AND WAGES (for participants age 16 and older who work)

1. What is your primary occupation? _ _ B4
   - 01 senior professional/technical worker (doctor, professor, lawyer, architect, engineer)
   - 02 junior professional/technical worker (midwife, nurse, teacher, editor, photographer)
   - 03 administrator/executive/manager (working proprietor, government official, section chief, department or bureau director, administrative cadre, village leader)
   - 04 office staff (secretary, office helper)
   - 05 farmer, fisherman, hunter
   - 06 skilled worker (foreman, group leader, craftsman)
   - 07 non-skilled worker (ordinary laborer, logger)
   - 08 army officer, police officer
   - 09 ordinary soldier, policeman
   - 10 driver
   - 11 service worker (housekeeper, cook, waiter, doorkeeper, hairdresser, counter salesperson, launderer, child care worker)
   - 12 athlete, actor, musician
   - 13 other (specify: __________)
   - -9 unknown

2. What is your employment position in this occupation? _ B5
   1 self-employed, owner-manager with employees
   2 self-employed, independent operator with no employees (includes farmer)
   3 works for another person or enterprise (includes small-, medium-, and large-scale collective enterprise, farm, and private enterprise) as a permanent employee
   4 contractor with other people or enterprise
   5 temporary worker
   6 paid family worker
   7 unpaid family worker
   8 other (specify: __________)
   9 unknown

3. What type of work unit is this? _ _ B6
   01 government department
   02 state service/institute
   03 state-owned enterprise
   04 small collective enterprise (such as township-owned)
   05 large collective enterprise (such as owned by county, city, province)
   06 family contract farming
   07 private, individual enterprise
   08 three-capital enterprise (owned by foreigners, overseas Chinese and joint venture)
   09 other (specify: __________)
   - 9 unknown

4. How many employees does this work unit have? _ B7
   1 < 20
   2 20-100
   3 >100
   9 unknown

5. In 2014, for how many months did you work at this occupation? _ _ C3
   * If "unknown," record -9.

6. For how many days in a week, on the average, did you work? _ C5
   * If "unknown," record 9.

7. For how many hours in a day, on the average, did you work? _ _ C6
   * If "unknown," record -9.

8. During the past week, for how many hours did you work? _ _ _ C7
   * If "unknown," record -99.

9. Were you paid a regular wage in 2014? _ C7b
   0 No(skip to question 11)
   1 yes

10. On the average, what was your monthly wage/salary in 2014, including subsidies? (yuan) _ _ _ _ _ C8
   * If "unknown," record -9999.

11. Did you receive a bonus in 2014(including monthly bonus, quarterly bonus,_ year-end bonus, holiday bonus, and other bonus)? _ I18
   0 no (skip to the next section)
   1 yes
   9 unknown (skip to the next section)

12. In 2014, what was the total value of all bonuses for the entire year? (yuan) _ _ _ _ _ I19
   * If "unknown," record -9999.

IV. SECONDARY OCCUPATION AND WAGES (for participants age 16 and older who work)

1. Do you have a secondary occupation? _ B9a
   - 0 no (skip to the next section)
   - 1 yes
   - 9 unknown (skip to the next section)

2. What is your employment position in this occupation? _ B10
   - 1 self-employed, owner-manager with employees
   - 2 self-employed, independent operator with no employees (includes farmer)
   - 3 works for another person or enterprise (includes small-, medium-, and large-scale collective enterprise, farm, and private enterprise) as a permanent employee
   - 4 contractor with other people or enterprise
   - 5 temporary worker
   - 6 paid family worker
   - 7 unpaid family worker
   - 8 other (specify: __________)
   - 9 unknown

3. In 2014, for how many months did you work at this occupation? _ _ C3a
   * If "unknown," record -9.

4. For how many days in a week, on the average, did you work? _ C5a
   * If "unknown," record 9.

5. For how many hours in a day, on the average, did you work? _ _ C6a
   * If "unknown," record -9.

6. During the past week, for how many hours did you work? _ _ _ C7a
   * If "unknown," record -99.

7. Were you paid a regular wage in 2014? _ C7c
   - 0 no(skip to question 9)
   - 1 yes

8. On the average, what was your monthly wage/salary in 2014, <u>including</u> subsidies? (yuan) _ _ _ _ _ C8a
   * If "unknown," record -9999.

9. Did you receive a bonus in 2014(including monthly bonus, quarterly bonus, year-end bonus, holiday bonus, and other bonus)? _ I18a
   - 0 no (skip to the next section)
   - 1 yes
   - 9 unknown (skip to the next section)

10. In 2014, what was the total value of all bonuses for the entire year? (yuan) _ _ _ _ _ I19a
    * If "unknown," record -9999.

V. HOME GARDENING (for participants age 6 and older)

1. Did you work in a household vegetable garden or orchard in 2014? _ D2a
   - 0 no (skip to the next section)
   - 1 yes

2. In 2014, for how many months did you engage in such work? _ _ D3a
   * If "unknown," record -9.

3. For how many days in a week, on the average, did you work? _ D3b
   * If "unknown," record 9.

4. For how many hours in a day, on the average, did you work? _ _ D3c
   * If "unknown," record -9.

VI. COLLECTIVE AND HOUSEHOLD FARMING (for participants age 6 and older)

1. Did you work on a collective farm or a household farm in 2014? _ E2a
   - 0 no (skip to the next section)
   - 1 yes

2. In 2014, for how many months did you work on a farm (collective or household)? _ _ E4a
   * If "unknown," record -9.

3. For how many days in a week, on the average, did you work? _ E4b
   * If "unknown," record 9.

4. For how many hours in a day, on the average, did you work? _ _ E4c
   * If "unknown," record -9.

5. What kind of farming business is this? _ E5
   - 1 collective farm
   - 2 household farm (skip to Question 10)
   - 3 both collective and household

6. Did you receive money from the collective in 2014? _ E6
   - 0 no (skip to Question 8)
   - 1 yes
   - 9 unknown (skip to Question 8)

7. How much money did you receive? (yuan) _ _ _ _ _ E7
   * If "unknown," record -9999.

8. Did you receive farm produce and/or other items, such as durable goods, from the collective in 2014? _ E8
   - 0 no (skip to Question 10)
   - 1 yes
   - 9 unknown (skip to Question 10)

9. How much money were these farm produce and/or other items you received worth? (yuan) _ _ _ _ E9
   * If "unknown," record -999.

10. Are you the household member primarily responsible for the household's farming activities? _ E10
    - 0 no
    - 1 yes

VII. RAISING LIVESTOCK/POULTRY(for participants age 6 and older)

1. Did you work raising livestock or poultry either on a collective or at home in 2014 _ F2a
   - 0 no (skip to the next section)
   - 1 yes

2. In 2014, for how many months did you work raising livestock or poultry? _ _ F4a
   * If "unknown," record -9.

3. For how many days in a week, on the average, did you work? _ F4b
   * If "unknown," record 9.

4. For how many hours in a day, on the average, did you work? _ _ F4c
   * If "unknown," record -9.

5. What kind of livestock- or poultry-raising business is this? _ F5
   - 1 collective
   - 2 household (skip to Question 10)
   - 3 both collective and household

6. Did you receive money from the collective in 2014? _ F6
   - 0 no (skip to Question 8)
   - 1 yes
   - 9 unknown (skip to Question 8)

7. How much money did you receive? (yuan) _ _ _ _ F7
   * If "unknown," record -999.

8. Did you receive livestock or poultry products from the collective in 2014? _ F8
   - 0 no (skip to Question 10)
   - 1 yes
   - 9 unknown (skip to Question 10)

9. How much money were these livestock or poultry products you received worth? (yuan) _ _ _ _ F9
   * If "unknown," record -999.

10. Are you the household member primarily responsible for the household's livestock or poultry business? _ F10
    - 0 no
    - 1 yes

VIII. COLLECTIVE AND HOUSEHOLD FISHING (for participants age 6 and older)

1. Did you work in fishing either on a collective or in a business operated by your household in 2014? _ G2a
   - 0 no (skip to the next section)
   - 1 yes

2. In 2014, for how many months did you work in fishing? _ _ G4a
   * If "unknown," record -9.

3. For how many days in a week, on the average, did you work? _ G4b
   * If "unknown," record 9.

4. For how many hours in a day, on the average, did you work? _ _ G4c
   * If "unknown," record -9.

5. What kind of fishing business is this? _ G5
   - 1 collective
   - 2 household (skip to Question 10)
   - 3 both collective and household

6. Did you receive money from the collective in 2014? _ G6
   0 no (skip to Question 8)
   1 yes
   9 unknown (skip to Question 8)

7. How much money did you receive? (yuan) _ _ _ _ G7
   * If “unknown,” record -999.

8. Did you receive fish or other goods from the collective in 2014? _ G8
   0 no (skip to Question 10)
   1 yes
   9 unknown (skip to Question 10)

9. How much money were these fish or goods you received worth? (yuan) _ _ _ _ G9
   * If “unknown,” record -999.

10. Are you the household member primarily responsible for the household’s fishing business? _ G10
   0 no
   1 yes

IX. SMALL HANDICRAFT AND SMALL COMMERCIAL HOUSEHOLD BUSINESS

(for participants age 6 and older)

1. Did you work in a small handicraft or small commercial business operated by your household in 2014(such as carpentry, shoe repair, housekeeping/child care service, tailoring, hairdressing, electrical appliance repair, restaurant, store, family child care, family hotel, family clinic, etc.)? _ H1c
   0 no (skip to the next section)
   1 yes

*** Ask Questions 4-8 about each business and record the answers in Table 1.**
* Be sure to classify each business the same way it was classified in the household questionnaire.
* If works in more than one business of the same type, such as tailoring and hairdressing (both are services), add together the amount of time worked in these businesses and record the total for this type in Table 1.

Table 1. Small Household Businesses

2<br>Business number<br>**H1d**	3<br>Business type	4<br>Did you work in this business in 2014?<br>0 no<br>1 yes<br>* If “no,” skip down to next item.<br>**H5a**	5<br>In 2014, for how many months did you work in this business?<br>* If “unknown,” record -9.<br>**H6**	6<br>For how many days in a week, on the average, did you work?<br>* If “unknown,” record 9.<br>**H7**	7<br>For how many hours in a day, on the average, did you work?<br>* If “unknown,” record -9.<br>**H8**	8<br>During the past week, for how many hours did you work?<br>* If “unknown,” record -99.<br>**H9**
1	Commerce	_	_ _	_	_ _	_ _ _
2	Service	_	_ _	_	_ _	_ _ _
3	Manufacturing	_	_ _	_	_ _	_ _ _
4	Peddler	_	_ _	_	_ _	_ _ _
5	Construction	_	_ _	_	_ _	_ _ _
6	Other(specify:_ ___)	_	_ _	_	_ _	_ _ _

X. OTHER SOURCES OF INCOME (for all participants)

1. Did you have any other cash income excluding cash from other household members in 2014? _ I100
   - 0 no (skip to Question 3)
   - 1 yes
   - 9 unknown (skip to Question 3)

2. How much money was it in 2014?* If unknown, record -9999. _ _ _ _ _ I101

3. Did you have any non-cash income (e.g. clothes, foods, etc) excluding those from other household members in 2014? _ I102
   - 0 no (skip to next section)
   - 1 yes
   - 9 unknown (skip to next section)

4. How much was it if you bought them from market in 2014? _ _ _ _ _ I103

XI. TIME ALLOCATION FOR HOME ACTIVITIES (for participants age 6 and older)
*** Ask Questions 2-3 about each activity and record the answers in Table 2.**

Table 2. Home Activities (Household Chores)

1<br>Activity type	2<br>During the past week, did you do this chore?<br>0 no 1 yes 9 unknown<br>* If "no" or "unknown," skip down to next item.	3<br>How much time did you spend per day, on average? (minutes)<br>* If does not know the exact time, record -99.
Buy food for your household	_ K2	_ _ _ K3 *if done on the way to/from school/work, record -88
Prepare and cook food for your household	_ K4	_ _ _ K5
Wash clothes with washing machine	_ K6a	_ _ _ K6b
Wash and iron clothes by hand	_ K6	_ _ _ K7
Clean the house	_ K7b	_ _ _ K7c

XII. CARE OF CHILDREN UNDER AGE 6 (for participants age 6 and older)

1. During the past week, did you take care of children under 6 in your household? _ K12
   - 0 no (skip to Question 3)
   - 1 yes
   - 9 unknown (skip to Question 3)

2. How much time did you spend taking care of the children by feeding, bathing, dressing, holding, or watching them during the past week? (hours) _ _ _ K13
   * Time should be counted even if doing something else while caring for the children, such as cooking a meal or washing clothes. If does not know the exact time, record -99.

3. Did you take care of children under age 6 for another household during the past week? _ K13b
   - 0 no (skip to the next section)
   - 1 Yes
   - 9 unknown (skip to the next section)

4. How much time did you spend taking care of children under age 6 for another household during the past week? (hours) _ _ _ K13c
   * If does not know the exact time, record -99.

XIII. CHILD CARE OUTSIDE THE HOME (for children under age 6. Ask their parents or guardians)

1. During the past week, were you taken care of by people who do not live in your household? _ K14a
   - 0 no (skip to Question 4)
   - 1 yes
   - 9 unknown (skip to Question 4)
2. Where did the care take place?

(1)	In your home	0 no	1 yes	9 unknown	_ K15
(2)	In the home of your paternal grandparents	0 no	1 yes	9 unknown	_ K16
(3)	In the home of your maternal grandparents	0 no	1 yes	9 unknown	_ K17
(4)	In the home of other relatives	0 no	1 yes	9 unknown	_ K18
(5)	In the home of neighbors	0 no	1 yes	9 unknown	_ K19
(6)	In a neighborhood or private child care center	0 no	1 yes	9 unknown	_ K20
(7)	In a state child care center	0 no	1 yes	9 unknown	_ K21
(8)	In a child care center run by a work unit	0 no	1 yes	9 unknown	_ K22
(9)	At a preschool managed by a primary school	0 no	1 yes	9 unknown	_ K23
(10)	At a nursery school	0 no	1 yes	9 unknown	_ K24
(11)	Other (specify: __________)	0 no	1 yes	9 unknown	_ K25

3. During the past week, for how many hours were you taken care of by people who do not live in your household? (hours) _ _ _ K42a
   * If does not know the exact time, record -99.

4. For how many days in a typical week are you taken care of by people who do not live in your household? (days) _ K42
   * If does not know the exact time, record 9.

5. For how many hours in a typical day are you taken care of by people who do not live in your household? (hours) _ _ K41
   * If for the entire day, record 24 hours. If does not know the exact time, record -9.

XIV. SMOKING (for participants age 12 and older)

1. Have you ever smoked cigarettes (including hand-rolled or device-rolled)? _ U25
   - 0 never smoked (skip to the next section)
   - 1 yes
   - 9 unknown (skip to the next section)

2. How old were you when you started to smoke? (years) _ _ _ U26
   * If “unknown,” record -99.

3. Do you still smoke cigarettes now? _ U27
   - 0 no (skip to Question 5)
   - 1 yes
   - 9 unknown (skip to Question 5)

4. How many cigarettes do you smoke per day? _ _ U28
   * If “unknown,” record -9.
   *** Skip to the next section.**

5. How long ago did you stop smoking? (months) _ _ _ U29
   * If “unknown,” record -99.

XV. ALCOHOL CONSUMPTION (for participants age 15 and older)

1. In 2014, did you drink beer or any other alcoholic beverage? _ U40
   - 0 no (skip to the next section)
   - 1 Yes
   - 9 unknown (skip to the next section)

2. How often did you drink beer or any alcoholic beverage? _ U41
   - 1 almost every day
   - 2 3-4 times a week
   - 3 once or twice a week
   - 4 once or twice a month
   - 5 no more than once a month
   - 9 Unknown

*** Ask Questions 4-5 about each type of alcohol and record the answers in Table 3.**

Table 3. Alcohol Consumption

3<br>Alcohol type	4<br>Do you drink this type of alcohol?<br>0 no 1 yes 9 unknown<br>* If "no" or "unknown," skip down to next item.	5<br>How much do you drink each week?<br>* If "unknown," record -9.
Beer	_ U42a	_ _ U42 (bottle)
Grape wine (including various colored wines, rice wine)	_ U43a	_ _ U43 (liang)
Liquor	_ U44a	_ _ U44 (liang)

XVI. PHYSICAL ACTIVITIES (for children under age 6, ask their parents or guardians)

1. How many hours each day do you usually sleep, including daytime and nighttime? (hours) _ _ U324
   * If "unknown," record -9.

2. Usually, do you do any physical exercises (e.g., running, using playground equipment, playing soccer or other sports) in preschool facilities, athletic schools, or at home? _ U90
   - 0 no (skip to Table 4)
   - 1 Yes
   - 9 unknown (skip to Table 4)

3. How many hours do you spend doing physical exercises each week? _ _ U91
   * If "unknown," record -9.

*** Ask Questions 5-7 about each activity and record the answers in Table 4.**

Table 4. Sedentary Activities for Children under Age 6

4<br>Activity type	5<br>Do you participate in this activity?<br>0 no 1 yes 9 unknown<br>* If "no" or "unknown," skip down to next item.	6/7<br>How much time do you spend during a typical day? (hours:minutes)<br>* If "unknown," record -9:99.	
		Monday - Friday	Saturday - Sunday
TV	_ U339	_ _ : _ _ U340	_ _ : _ _ U341
Videotapes, VCDs, DVDs	_ U342	_ _ : _ _ U343	_ _ : _ _ U344
Watching movies and videos online or on a smartphone	_ U508	_ _ : _ _ U509	_ _ : _ _ U510
Video games	_ U345	_ _ : _ _ U346	_ _ : _ _ U347
Surfing the internet	_ U410	_ _ : _ _ U411	_ _ : _ _ U412
Participating in chat rooms including QQ and WeChat	_ U413	_ _ : _ _ U414	_ _ : _ _ U415
Playing computer/smartphone games, etc.	_ U416	_ _ : _ _ U417	_ _ : _ _ U418
Reading (books, newspapers and magazines), writing, drawing	_ U351	_ _ : _ _ U352	_ _ : _ _ U353
Toy cars, puppets, board games	_ U96a	_ _ : _ _ U249	_ _ : _ _ U250

* **Ask Questions 8-11 about each activity and record the answers in Table 5.**

Table 5. Physical Activities for Children under 6

8<br>Activity type	9<br>Do you participate in this activity <u>before or after school or on the weekend</u>?<br>0 no 1 yes 9 unknown<br>* If "no" or "unknown," skip down to next item.	10/11<br>How much time do you spend during a typical day? (hours:minutes)<br>* If "unknown," record -9:99.	
		Monday - Friday	Saturday - Sunday
Martial arts (Kung Fu, etc.)	_ U145	_ _ : _ _ U327	_ _ : _ _ U328
Gymnastics, dancing, acrobatics	_ U149	_ _ : _ _ U329	_ _ : _ _ U330
Track and field (running, etc.), swimming	_ U147	_ _ : _ _ U331	_ _ : _ _ U332
Walking	_ U147b	_ _ : _ _ U331b	_ _ : _ _ U332b
Soccer, basketball, tennis	_ U151	_ _ : _ _ U333	_ _ : _ _ U334
Badminton, volleyball	_ U153	_ _ : _ _ U335	_ _ : _ _ U336
Other (ping pong, Tai Chi, etc.)	_ U155	_ _ : _ _ U337	_ _ : _ _ U338

XVII. PHYSICAL ACTIVITIES (for participants age 6 and older who are in school)

1. How many hours each day do you usually sleep, including daytime and nighttime? (hours) _ _ U324
   * If "unknown," record -9.

2. Do you participate in any physical exercises <u>before or after school or on the weekend</u>, including relatively intense physical exercises, such as volleyball, soccer, badminton, and long distance running? _ U98
   - 0 no (skip to Table 7)
   - 1 Yes
   - 9 unknown (skip to Table 7)

3. How many times do you participate in any physical exercises <u>before or after school or on the weekend</u> each week? _ _ U99a
   * If "unknown," record -9.

4. On average, for how long do you participate in these physical exercises each time? (hours:minutes). **_ _:_ _** U99b
* If "unknown," record -9:99

*** Ask Questions 6-8 about each activity and record the answers in Table 6.**

Table 6. Physical Activities for Children Age 6 and Older Who Are in School: Activities Before or After School or on the Weekend

5<br>Activity type	6<br>Do you participate in this activity <u>before or after school or on the weekend</u>?<br>0 no 1 yes 9 unknown<br>* If "no" or "unknown," skip down to next item.	7/8<br>How much time do you spend during a typical day? (hours:minutes)<br>* If "unknown," record -9:99.	
		Monday - Friday	Saturday - Sunday
Martial arts (Kung Fu, etc.)	_ U145	_ _ : _ _ U327	_ _ : _ _ U328
Gymnastics, dancing, acrobatics	_ U149	_ _ : _ _ U329	_ _ : _ _ U330
Track and field (running, etc.), swimming	_ U147	_ _ : _ _ U331	_ _ : _ _ U332
Walking	_ U147b	_ _ : _ _ U331b	_ _ : _ _ U332b
Soccer, basketball, tennis	_ U151	_ _ : _ _ U333	_ _ : _ _ U334
Badminton, volleyball	_ U153	_ _ : _ _ U335	_ _ : _ _ U336
Other (ping pong, Tai Chi, etc.)	_ U155	_ _ : _ _ U337	_ _ : _ _ U338

*** Ask Questions 10-12 about each activity and record the answers in Table 7.**

Table 7. Sedentary Activities for Children Age 6 and Older Who Are in School: Activities Before or After School or on the Weekend

9<br>Activity type	10<br>Do you participate in this activity <u>before or after school or on the weekend</u>?<br>0 no 1 yes 9 unknown<br>* If "no" or "unknown," skip down to next item	11/12<br>How much time do you spend during a typical day? (hours:minutes)<br>* If "unknown," record -9:99.	
		Monday - Friday	Saturday - Sunday
TV	_ U339	_ _ : _ _ U340	_ _ : _ _ U341
Videotapes, VCDs, DVDs	_ U342	_ _ : _ _ U343	_ _ : _ _ U344
Watching movies and videos online or on a smartphone	_ U508	_ _ : _ _ U509	_ _ : _ _ U510
Video games	_ U345	_ _ : _ _ U346	_ _ : _ _ U347
Surfing the internet	_ U410	_ _ : _ _ U411	_ _ : _ _ U412
Participating in chat rooms including QQ and WeChat	_ U413	_ _ : _ _ U414	_ _ : _ _ U415
Playing computer/smartphone , games, etc.	_ U416	_ _ : _ _ U417	_ _ : _ _ U418
Doing homework	_ U220	_ _ : _ _ U273	_ _ : _ _ U274
Extracurricular reading (books, newspapers and magazines), writing, drawing	_ U351	_ _ : _ _ U352	_ _ : _ _ U353
Toy cars, puppets, board games	_ U96a	_ _ : _ _ U249	_ _ : _ _ U250

13. Do you have any physical exercise class <u>in school</u>? _ U108
    - 0 no (skip to Table 9)
    - 1 Yes
    - 9 unknown (skip to Table 9)

14. How many times do you participate in physical exercises <u>in school</u> (in class or at recess) each week? _ _ U109
    * If "unknown," record -9.

15. On average, for how long do you participate in these physical exercises each time? (hours: minutes) **_ _:_ _** U109a
* If "unknown," record -9:99.

*** Ask Questions 16-18 about each activity and record the answers in Table 8.**

Table 8. Physical Activities for Children Age 6 and Older Who Are in School: Activities in School

16<br>Activity type	17<br>Do you participate in this activity in school?<br>0 no 1 yes 9 unknown<br>* If "no" or "unknown," skip down to next item	18<br>How much time do you spend each week?(hours:minutes)<br>* If "unknown," record -9:99
Martial arts (Kung Fu, etc.)	_ U221	_ _ : _ _ U285
Gymnastics, dancing, acrobatics	_ U110	_ _ : _ _ U111
Track and field (running, etc.), swimming	_ U114	_ _ : _ _ U115
Walking	_ U114b	_ _ : _ _ U115b
Soccer, basketball, tennis	_ U222	_ _ : _ _ U286
Badminton, volleyball	_ U223	_ _ : _ _ U287
Other (ping pong, Tai Chi, etc.)	_ U224	_ _ : _ _ U288

*** Ask Questions 19-21 about each transportation type and record the answers in Table 9.**

Table 9. Transportation To and From School for Children Age 6 and Older Who Are in School

19<br>Transportation method	20<br>Do you travel to and from school this way?<br>0 no 1 yes 9 unknown<br>* If "no" or "unknown," skip down to next item.	21<br>How long does a round trip take? (hours:minutes)<br>* If "unknown," record -9:99.
Walk	_ U128	_ _ : _ _ U129
Bicycle [(pedaled)]	_ U126	_ _ : _ _ U127
Bicycle (passenger)	_ U126b	_ _ : _ _ U127b
Bus, subway	_ U124	_ _ : _ _ U125
Car, taxi, motorcycle	_ U325	_ _ : _ _ U326

XVIII. PHYSICAL ACTIVITIES (for participants age 6 and older who are not in school)

1. How many hours each day do you usually sleep, including daytime and nighttime? (hours) _ _ U324
* If "unknown," record -9.

2. Do you participate in any physical exercises or outdoor games? _ U98
   0 no (skip to Table 11)
   1 Yes
   9 unknown (skip to Table 11)

3. How many times do you participate in any physical exercises or outdoor games each week? _ _ U99a
* If does not participate in these activities, record 00.
If "unknown," record -9.

4. On average, for how long do you participate in these physical exercises each time? (hours:minutes) _ _ U99b
* If "unknown," record -9:99.

*** Ask Questions 6-8 about each activity and record the answers in Table 10.**

Table 10. Physical Activities

5<br>Activity type	6<br>Do you participate in this activity?<br>0 no 1 yes 9 unknown<br>*** If "no" or "unknown," skip down to next item.**	7/8<br>How much time do you spend during a typical day? (hours:minutes)<br>*** If "unknown," record -9:99.**	
		Monday - Friday	Saturday - Sunday
Martial arts (Kung Fu, etc.)	_ U145	_ _ : _ _ U327	_ _ : _ _ U328
Gymnastics, dancing, acrobatics	_ U149	_ _ : _ _ U329	_ _ : _ _ U330
Track and field (running, etc.), swimming	_ U147	_ _ : _ _ U331	_ _ : _ _ U332
Walking	_ U147b	_ _ : _ _ U331b	_ _ : _ _ U332b
Soccer, basketball, tennis	_ U151	_ _ : _ _ U333	_ _ : _ _ U334
Badminton, volleyball	_ U153	_ _ : _ _ U335	_ _ : _ _ U336
Other (ping pong, Tai Chi, etc.)	_ U155	_ _ : _ _ U337	_ _ : _ _ U338

*** Ask Questions 10-12 about each activity and record the answers in Table 12.**

Table 12. Sedentary Activities

9<br>Activity type	10<br>Do you participate in this activity?<br>0 no 1 yes 9 unknown<br>* If "no" or "unknown," skip down to next item.	11/12<br>How much time do you spend during a typical day? (hours:minutes)<br>* If "unknown," record -9:99.	
		Monday - Friday	Saturday - Sunday
TV	_ U339	_ _ : _ _ U340	_ _ : _ _ U341
Videotapes, VCDs, DVDs	_ U342	_ _ : _ _ U343	_ _ : _ _ U344
Watching movies and videos online or on a smartphone	_ U508	_ _ : _ _ U509	_ _ : _ _ U510
Video games	_ U345	_ _ : _ _ U346	_ _ : _ _ U347
Surfing the internet	_ U410	_ _ : _ _ U411	_ _ : _ _ U412
Participating in chat rooms including QQ and WeChat	_ U413	_ _ : _ _ U414	_ _ : _ _ U415
Playing computer/smartphone games, etc.	_ U416	_ _ : _ _ U417	_ _ : _ _ U418
Reading (books, newspapers and magazines), writing, drawing	_ U351	_ _ : _ _ U352	_ _ : _ _ U353
Other sedentary activities	_ U351a	_ _ : _ _ U352a	_ _ : _ _ U353a

*** Ask Questions 14-15 about each transportation type for adults who work and record the answers in Table 12.**

Table 12. Transportation to and from Work

13<br>Transportation method	14<br>Do you travel to and from work this way?<br>0 no 1 yes 9 unknown<br>* If "no" or "unknown," skip down to next item.	15<br>How long does a <u>round trip</u> take? (hours:minutes)<br>* If "unknown," record -9:99.
Walk	**_ U128**	**_ _ : _ _ U129**
Bicycle (pedaled)	**_ U126**	**_ _ : _ _ U127**
Bicycle (passenger)	**_ U126b**	**_ _ : _ _ U127b**
Bus, subway	**_ U124**	**_ _ : _ _ U125**
Car, taxi, motorcycle	**_ U325**	**_ _ : _ _ U326**

*** Ask Question 16 for adults who work:**

16. How much time do you spend doing each of these types of physical activities during work time in a typical week? (hours:minutes)
*** If "none," record 00:00. If "unknown," record -9:99.**
    (1) Light physical activities (e.g., sedentary job, job with some standing and sitting, office work, watch smith, counter salesperson, lab technician) **_ _ : _ _** U140
    (2) Moderate physical activities (e.g., driver, electrician) **_ _ : _ _** U141
    (3) Heavy physical activities (e.g., farmer, athlete, dancer, steel worker, lumber worker, mason) **_ _ : _ _** U142

XIX. ACCESS TO THE INTERNET (for participants age 6 and older)

1. Can you access to the internet? _ U354
    0 no (skip to the next section)
    1 yes
    9 unknown (skip to the next section)

2. Where can you access to the internet?

1	internet cafe	0 no	1 yes	9 unknown	_ U419
2	at home	0 no	1 yes	9 unknown	_ U427
3	at friend's or relative's home	0 no	1 yes	9 unknown	_ U428
4	in school	0 no	1 yes	9 unknown	_ U429
5	in work place	0 no	1 yes	9 unknown	_ U429a
6	in stores or other public areas	0 no	1 yes	9 unknown	_ U429b
7	With smartphone/IPad or similar devices	0 no	1 yes	9 unknown	_ U429c

3. Do you ever go to an internet cafe? _ U355
    0 no (skip to the next section)
    1 Yes
    9 unknown (skip to the next section)

4. Which of these things do you usually do at an internet café?

1	Surf the internet	0 no	1 yes	9 unknown	_ U356
2	Participate in chat rooms	0 no	1 yes	9 unknown	_ U357
3	Play games	0 no	1 yes	9 unknown	_ U358
4	Check emails/messages	0 no	1 yes	9 unknown	_ U359a
5	Other (specify: __________)	0 no	1 yes	9 unknown	_U359

XX. BODY SHAPE AND MASS MEDIA (for participants age 6 to 17)

1. Look at these body shape pictures. Which one looks most like you? _ U200
   * Shuffle all pictures first. Then show them to the child and ask him/her to choose one. Record the number from the back of the picture.

2. Look at these pictures again. Which one do you want your body to look like? _ U201
   * Collect all pictures, shuffle, and show them to the child. Ask him/her to choose one. Record the number from the back of the picture.

3. Look at these pictures again. Which one do you think is the healthiest? _ U201a
   * Collect all pictures, shuffle, and show them to the child. Ask him/her to choose one. Record the number from the back of the picture.

4. <u>During the past 3 months</u>, how many times have you eaten at a Western fast food restaurant, such as McDonald's or Kentucky Fried Chicken? _ _ U367
   * If "unknown," record -9.

5. Do you think you are now underweight, normal or overweight? _ U203
   1 underweight; 3 overweight
   2 normal; 9 unknown

6. Were you on a diet in 2014? "On a diet" means changing your normal eating habits to lose or gain weight. _ U202
   0 no; 2 yes, on a diet to lose weight
   1 yes, on a diet to gain weight; 9 unknown

7. Do you think you have too little, just the right amount, or too much physical activity? Physical activity refers to sports or activities that increase your heart rate or make you sweat. _ U204
   1 too little; 3 too much
   2 just the right amount; 9 unknown

8. Does your family ever ask you to engage in more physical activity, less physical activity, or don't they care? _ U368
   0 no, don't care; 2 yes, less
   1 yes, more; 9 unknown

9. Do you have a TV (in working order) at home? _ U205
   0 no (skip to the next section)
   1 yes

10. Do you have a TV (in working order) in your bedroom? _ U369
    0 no
    1 yes

11. How many days per week do you watch TV with one or both of your parents? _ U447
    0 very seldom (< 1 times/month); 3 Often (3-4 times/wk)
    1 Seldom (1-3 times/month); 4 very often ($\geq$ 5times/wk)
    2 Sometimes(1-2 times/wk); 9 unknown

12. How often do your parents tell you that something you've seen somebody do on TV is not OK? _ U448
    0 very seldom (< 1 times/month); 3 Often (3-4 times/wk)
    1 Seldom (1-3 times/month); 4 very often ($\geq$ 5times/wk)
    2 Sometimes(1-2 times/wk); 9 unknown

13. How often do your parents tell you that something on TV is not real? _ U449
    0 very seldom (< 1 times/month); 3 Often (3-4 times/wk)
    1 Seldom (1-3 times/month); 4 very often ($\geq$ 5times/wk)
    2 Sometimes(1-2 times/wk); 9 unknown

14. Does your family have rules about <u>how long</u> you can watch TV? _ U206c
    0 very seldom (< 1 times/month); 3 Often (3-4 times/wk)
    1 Seldom (1-3 times/month); 4 very often ($\geq$ 5times/wk)
    2 Sometimes(1-2 times/wk); 9 unknown

15. Does your family have rules about what kinds of TV shows you can watch? _ U206d

0	very seldom (< 1 times/month)	3	Often (3-4 times/wk)
1	Seldom (1-3 times/month)	4	very often (≥ 5times/wk)
2	Sometimes(1-2 times/wk)	9	unknown

16. Which TV programs do you like best? _ U209
Second best? _ U210

0 no preference
1 sports
2 pop music (such as MTV), popular or non-traditional dance
3 drama
4 news
5 economy/geography/history/politics
6 TV series/movies
7 cartoons

17. Do you eat snacks while watching TV? _ U371a

0	very seldom (< 1 times/month)	3	Often (3-4 times/wk)
1	Seldom (1-3 times/month)	4	very often (≥ 5times/wk)
2	Sometimes(1-2 times/wk)	9	unknown

18. Do you watch TV when you are eating a meal? _ U372a

0	very seldom (< 1 times/month)	3	Often (3-4 times/wk)
1	Seldom (1-3 times/month)	4	very often (≥ 5times/wk)
2	Sometimes(1-2 times/wk)	9	unknown

19. Do you ask your parents to buy the kind of food or drinks you see on TV commercials? _ U213b

0	very seldom (< 1 times/month)	3	Often (3-4 times/wk)
1	Seldom (1-3 times/month)	4	very often (≥ 5times/wk)
2	Sometimes(1-2 times/wk)	9	unknown

20. Do your parents buy them for you? _ U214c

0	very seldom (< 1 times/month)	3	Often (3-4 times/wk)
1	Seldom (1-3 times/month)	4	very often (≥ 5times/wk)
2	Sometimes(1-2 times/wk)	9	unknown

21. Do you buy for yourself the kind of food or drinks you see on TV commercials? _ U373a

0	very seldom (< 1 times/month)	3	Often (3-4 times/wk)
1	Seldom (1-3 times/month)	4	very often (≥ 5times/wk)
2	Sometimes(1-2 times/wk)	9	unknown

XXI. ACTIVITIES OF DAILY LIVING(for adults age 55 and older)

We want to understand the various life difficulties caused by health and physical limitations.

*** Ask Question 2 about each activity and record the answers in Table 13.**

Table 13. Activities of Daily Living I

1<br>Activity type	2<br>Do you have any difficulty doing this?<br>1 no difficulty<br>2 have some difficulty, but can still do it<br>3 need help to do it<br>4 cannot do it at all<br>9 unknown
Running a kilometer	**_ U157**
Walking a kilometer	**_ U158**
Walking 200 meters	**_ U159**
Walking across a room	**_ U160**
Sitting continuously for two hours	**_U161**
Standing up after sitting for a long time	**_ U162**
Climbing one staircase	**_ U163**
Climbing a few stairs without stopping	**_ U164**
Lifting or raising a 5-kilogram bag<br>(such as a bag of flour, rice, or other miscellaneous items)	**_ U165**
Squatting down, kneeling down, or bending over	**_ U166**
Putting on your clothes	**_ U171**
Combing your hair	**_ U172**
Using the toilet	**_ U173**

*** Ask Questions 4-5 about each activity and record the answers in Table 14.**

Table 14. Activities of Daily Living II

3<br>Activity type	4<br>Do you have any difficulty doing this?<br>1 no difficulty (skip down to next item)<br>2 have some difficulty, but can still do it (skip down to next item)<br>3 need help to do it<br>4 cannot do it at all<br>9 unknown	5<br>If you need help, who helps you?<br>1 spouse<br>2 other family member<br>3 friend, relative or neighbor<br>4 health worker<br>5 other people<br>9 unknown
Bathing yourself	**_ U167**	**_ U168**
Eating by yourself	**_ U169**	**_ U170**

*** Ask Question 7 about each activity and record the answers in Table 15.**

Table 15. Activities of Daily Living III

6<br>Activity type	7<br>Does your health condition or physical strength make it difficult for you to do this?<br>1 no difficulty<br>2 have some difficulty, but can still do it<br>3 need help to do it<br>4 cannot do it at all<br>9 unknown
Shop (buying food, clothes, etc.) without others' help	**_ U174**
Cook without others' help	**_ U175**
Use public transportation to go places where it is too far to walk	**_ U176**
Manage your money (record your income and expenses, etc.) without others' help	**_ U177**
Use the telephone without others' help	**_ U178**

XXII. MEMORY TEST (for adults age 55 and older)

1. How is your memory? _ U179
   1 very good
   2 good
   3 OK
   4 bad
   5 very bad
   9 unknown

2. In the past twelve months, how has your memory changed? _ U180
   1 improved
   2 stayed the same
   3 deteriorated
   9 unknown

3. Now let's do a memory test. I'll read a few words and ask you to repeat them. There are quite a few words. It's hard for most people to remember all of them. Are you ready? Let's begin: _ _ U181
   House, wood, cat, table, night, needle, steamed bread, door, bridge, bed.

   * Read the words slowly and in a plain tone, approximately two seconds per word. Let the respondent think before he/she repeats, but not more than two minutes. Record the words and fill in the number of correct answers in the boxes.
   * If cannot remember, record 00. If does not answer, record -9.
   *** If refuses to answer, skip to the next section.**

4. Please count backward from 20 to 1. _ U185
   * If does not get it right the first time, try again.
   1 correct the first time
   2 correct the second time
   3 incorrect both times
   9 unknown

5. Ask the respondent the following questions:
   * If adds 7 instead of subtracts 7, repeat the question.
   *** If answer is correct, continue. Otherwise, skip to Question 6.**

(1)	How much is 100 minus 7?	0 incorrect	1 correct (93)	9 unknown	_ U187
(2)	Subtract 7 again. What is the result?	0 incorrect	1 correct (86)	9 unknown	_ U188
(3)	Subtract 7 again. What is the result?	0 incorrect	1 correct (79)	9 unknown	_ U189
(4)	Subtract 7 again. What is the result?	0 incorrect	1 correct (72)	9 unknown	_ U190
(5)	Subtract 7 again. What is the result?	0 incorrect	1 correct (65)	9 unknown	_ U191

6. I read a list of words to you just now. Now please repeat those words again. _ _ U192
   * Let the respondent think before he/she repeats, but no more than two minutes. Do not read the words again. Record the words and fill in the number of correct answers in the boxes. (The words were: House, wood, cat, table, night, needle, steamed bread, door, bridge, bed.)
   * If cannot remember, record 00. If does not answer, record -9.

XXIII. MEDICAL INSURANCE (for all participants)

1. Do you have medical insurance? _ M1
   0 no (skip to Question 8)
   1 yes
2. Which of the following types of medical insurance do you have?

(0)	Commercial medical insurance	0 no	1 yes	9 unknown	_ M3a_0
(1)	Government (Free)medical insurance	0 no	1 yes	9 unknown	_ M3a_1
(2)	Urban employee basic medical insurance	0 no	1 yes	9 unknown	_ M3a_12
(3)	Urban resident basic medical insurance	0 no	1 yes	9 unknown	_ M3a_13
(4)	Rural newly cooperative basic medical insurance	0 no	1 yes	9 unknown	_ M3a_4
(9)	Other (specify: __________)	0 no	1 yes	9 unknown	_ M3a_8

*** If more than one type of insurance, ask Questions 3-7 about the primary type (most frequently used).**

3. What is your monthly contribution to this insurance? (yuan)? _ _ _ M2a
   *If unknown, record -99.

4. Do you buy any supplementary medical insurance? _ M2b
   0 no (skip to question 6)
   1 Yes
5. What is your monthly contribution to this supplementary medical insurance? _ _ _ M2c
   * If unknown, record -99.
6. Does your employer buy any supplementary medical insurance for you? _ M2d
   0 no (skip to the next section)
   1 Yes
7. What is your monthly contribution to this supplementary medical insurance? _ _ _ M2e
   * If unknown, record -99.
   End for those who answered 2-7 and skip to next section.

8. Why do you have no medical insurance? _ M2f
   1 I do not need medical insurance because I am healthy.
   2 It is not worth because insurance reimburses only small amount of total medical costs.
   3 The premium is too high for me to afford
   4 Other reasons:_____________________________

XXIV. USE OF HEALTH CARE AND MEDICAL SERVICES (for all participants)

1. During the past 4 weeks, have you been sick or injured? Have you suffered from a chronic or acute disease? _ M23
   0 no
   1 yes
   9 unknown
2. Did you have any of these symptoms during the past 4 weeks (including today)?

(1)	Fever, sore throat, cough	0 no	1 yes	9 unknown	_ M24b_1
(2)	Diarrhea	0 no	1 yes	9 unknown	_ M24b_2
(2a)	Stomachache	0 no	1 yes	9 unknown	_ M24b_2a
(2b)	Asthma	0 no	1 yes	9 unknown	_ M24b_2b
(3)	Headache, dizziness	0 no	1 yes	9 unknown	_ M24b_3
(4)	Joint pain, muscle pain	0 no	1 yes	9 unknown	_ M24b_4
(5)	Rash, dermatitis	0 no	1 yes	9 unknown	_ M24b_5
(6)	Eye/ear disease	0 no	1 yes	9 unknown	_ M24b_6
(7)	Heart disease/chest pain	0 no	1 yes	9 unknown	_ M24b_7
(8)	Other infectious disease (specify: __________)	0 no	1 yes	9 unknown	_ M24b_8
(9)	Other noncommunicable disease (specify: __________)	0 no	1 yes	9 unknown	_ M24b_9

*** If no symptoms, skip to Question 16. Otherwise, ask Questions 3-15 about the most recent illness. Then ask Question 16.**

3. How severe was the illness or injury? _ M25
   1 not severe
   2 somewhat severe
   3 quite severe

4. For how many days during the past 4 weeks were you unable to carry out normal activities due to this illness? _ _ M26a
   * If "unknown," record -9.

5. What did you do when you felt ill? _ M26b
   1 Self-care
   2 saw the local health worker (skip to Question 8)
   3 saw a doctor (clinic, hospital) (skip to Question 8)
   4 did not pay any attention
   9 unknown
6. How much money did you spend on the illness or injury? (yuan) _ _ _ _ M39
   * If insurance covered all expenses, record -888. If "unknown," record -999.

7. What percentage of these costs was paid by insurance or may be paid by insurance? (%) _ _ _ _ M39a
   * If does not have medical insurance, record -88. If "unknown," record -99.

8. Did you seek care from a formal medical provider during the past 4 weeks? _ M52
   0 no (skip to Question 16)
   1 yes

9. Where did you see a doctor? _ _ M27b

01	village clinic	09	city maternal and child hospital
02	private clinic	10	city hospital
03	work unit clinic	11	worker's hospital
04	other clinic	12	other hospital
05	town family planning service	14	at home
06	town hospital	15	other (specify: __________)
07	county maternal and child hospital	- 9	unknown
08	county hospital		

10. Was it an outpatient or inpatient visit? _ M28
    0 outpatient (skip to Question 12)
    1 inpatient

11. For how many days <u>during the past 4 weeks</u> were you or have you been hospitalized? _ _ M29
    * If "unknown," record -9.

12. How much did this treatment cost or has this treatment cost so far (including all registration fees, medicines, treatment fees, bed fees, etc.)? (yuan) _ _ _ _ _ M30
    * If insurance covers all expenses, record -8888. If "unknown," record -9999.

13. What percentage of these costs was paid by insurance or may be paid by insurance? (%) _ _ _ M31
    * If does not have medical insurance, record -88. If "unknown," record -99.

14. How much money was spent or has been spent on treating your illness or injury in addition to the costs mentioned above? (yuan) _ _ _ M38
    * If "unknown," record -99.

15. What was the doctor's diagnosis of your illness or injury? _ _ M40

00	no diagnosis	12	eye/ear/nose/throat/teeth disease
01	infectious/parasitic disease	13	digestive disease
02	heart disease	14	urinary disease
03	tumor	15	sexual dysfunction
04	respiratory disease	16	obstetrical/gynecological disease
05	injury	17	neonatal disease
06	alcohol poisoning	18	dermatological disease
07	endocrine disorder	19	muscular/rheumatological disease
08	hematological disease	20	genetic disease
09	mental/psychiatric disorder	21	old age/mid-life syndrome
10	mental retardation	22	other (specify: __________)
11	neurological disorder	- 9	Unknown

16. Did you visit a folk doctor in 2014? _ M40a
    0 no
    1 yes
    9 unknown

17. <u>During the past 4 weeks</u>, did you receive any preventive health service, such as health examination, eye examination, blood test, blood pressure screening, tumor screening? _ M47
    0 no (skip to the next section)
    1 yes
    9 unknown (skip to the next section)

*** If more than one service, ask Questions 18-21 about the one that had the highest cost.**

18. What service did you receive? _ _ M48

01	general physical examination	07	prenatal examination
03	blood test	08	postnatal examination
04	blood pressure screening	09	gynecological examination
05	tumor screening	10	other (specify: __________)
06	vision or hearing examination	- 9	unknown

19. Where did you receive this service? _ _ M49

01	village clinic	09	city maternal and child hospital
02	private clinic	10	city hospital
03	work unit clinic	11	worker's hospital
04	other clinic	12	other hospital
05	town family planning service	14	at home
06	town hospital	15	other (specify: __________)
07	county maternal and child hospital	- 9	unknown
08	county hospital		

20. How much did this service cost? (yuan) _ _ _ . _ M50
* If total cost was paid by medical insurance, record -88.8. If "unknown,"record -99.9.

21. What percentage of this cost was paid by insurance, or may be paid by insurance? (%) _ _ _ M51
* If does not have medical insurance, record -88. If "unknown," record -99.

XXV. DISEASE HISTORY (for participants age 6 and older)

1. Has a doctor ever told you that you suffer from high blood pressure? _ U22
   - 0 no (skip to Question 4)
   - 1 yes
   - 9 unknown (skip to Question 4)

2. For how many years have you had it? _ _ _ U23
   * If "unknown," record -99.

3. Are you currently taking anti-hypertension drugs? _ U24
   - 0 no
   - 1 yes
   - 9 unknown

4. Has a doctor ever told you that you suffer from diabetes? _ U24a
   - 0 no (skip to Question 7)
   - 1 yes
   - 9 unknown (skip to Question 7)

5. How old were you when the doctor told you this? (years) _ _ _ U24b
   * If "unknown," record -99.

6. Did you use any of these treatment methods?

(1)	Special diet	0 no	1 yes	9 unknown	_ U24c
(2)	Weight control	0 no	1 yes	9 unknown	_ U24d
(3)	Oral medicine	0 no	1 yes	9 unknown	_ U24e
(4)	Injection of insulin	0 no	1 yes	9 unknown	_ U24f
(5)	Chinese traditional medicine	0 no	1 yes	9 unknown	_ U24g
(6)	Home remedies	0 no	1 yes	9 unknown	_ U24h
(7)	Qi Gong (spiritual method)	0 no	1 yes	9 unknown	_ U24i

* For children younger than 18, skip to Question 20.

7. Has a doctor ever given you the diagnosis of myocardial infarction? _ U24j
   - 0 No (skip to Question 11)
   - 1 Yes
   - 9 Unknown (skip to Question 11)

8. How old were you when the doctor told you this? (years) _ _ _ U24j1
* If “unknown,” record -99.

9. Have you had this problem in the past year? _ U24j2
0 No
1 Yes
9 Unknown

10. How old were you when you had this problem the most recent time? (years) _ _ _ U24k
* If “unknown,” record -99.

11. Has a doctor ever given you the diagnosis of stroke or transient ischemic attack? _ U24l
0 no (skip to Question 16)
1 Yes
9 unknown (skip to Question 16)

12. How old were you when you were first diagnosed with stroke or transient ischemic attack? (years) _ _ _ U24t
* If “unknown,” record -99.

13. Do you know what type of stroke you had in the first time? _ U24u
0 No
1 Ischemic
2 Hemorrhagic

14. Have you had this problem in the past year? _ U24v
0 No
1 Yes
9 Unknown

15. How old were you when you had this problem the most recent time? (years) _ _ _ U24m
* If “unknown,” record -99.

16. Has a doctor ever given you the diagnosis of cancer? _ U24w
0 No (skip to Question 20)
1 Yes
9 Unknown (skip to Question 20)

17. How old were you when you were first diagnosed with cancer? (years) _ _ _ U24x
* If “unknown,” record -99.

18. Do you know what type of cancer you suffer from?

1	lung cancer	0 no	1 yes	9 unknown	_ U24y01
2	stomach cancer	0 no	1 yes	9 unknown	_ U24 y02
3	Hepatic carcinoma	0 no	1 yes	9 unknown	_ U24 y03
4	Esophageal cancer	0 no	1 yes	9 unknown	_ U24 y04
5	colon cancer	0 no	1 yes	9 unknown	_ U24 y05
6	breast cancer	0 no	1 yes	9 unknown	_ U24 y06
7	cervical cancer	0 no	1 yes	9 unknown	_ U24 y07
8	blood/lymph glands cancer	0 no	1 yes	9 unknown	_ U24 y08
9	testes/scrotum cancer	0 no	1 yes	9 unknown	_ U24 y09
10	melanoma	0 no	1 yes	9 unknown	_ U24 y10
11	skin (not melanoma) cancer	0 no	1 yes	9 unknown	_ U24 y11
12	brain cancer	0 no	1 yes	9 unknown	_ U24 y12
13	uterine cancer	0 no	1 yes	9 unknown	_ U24 y13
14	prostate cancer	0 no	1 yes	9 unknown	_ U24 y14
15	bone cancer	0 no	1 yes	9 unknown	_ U24 y15
16	other (please specify)	0 no	1 yes	9 unknown	_ U24 y16

19. How old were you when you were diagnosed with cancer the most recent time? (years) _ _ _ U24w2
* If “unknown,” record -99.

20. Do you have a history of bone fracture? _ U24n
  0 No (skip to Question 23)
  1 Yes
  9 Unknown (skip to Question 23)

21. How old were you when you had the first bone fracture? (years) _ _ _ U24o
  * If "unknown," record -99.

22. How many times has this happened (including the first time)? _ _ U24p
  * If "unknown," record -9.

23. Has a doctor ever told you that you suffered from asthma? _ U24q
  0 No
  1 Yes
  9 Unknown

24. Have you had wheezing or whistling in the chest in the past year? _ U24r
  0 No (skip to Question 26)
  1 Yes
  9 Unknown (skip to Question 26)

25. For how many years have you had it? _ _ U24s
  * If "unknown," record -9.

26. How do you rate the quality of your life at present? _ U420
  1 Very good 4 Bad
  2 Good 5 Very bad
  3 Fair 9 Unknown

27. Right now, how would you describe your health compared to that of other people your age? _ U48a
  1 Very good 4 Bad
  2 Good 5 Very bad
  3 Fair 9 Unknown

*** Ask Questions 29-30 about psychological wellbeing and record the answers in Table 16 (for participants age 50 and older).**

Table 16. Psychological wellbeing

28<br>Item No.	29<br>Statement<br><br>Please use 1-5 to describe if you strongly disagree, somewhat disagree, neutral, somewhat agree, or strongly agree with this statement?	30<br>1 strongly disagree<br>2 disagree<br>3 neutral<br>4 agree<br>5 strongly agree<br>9 unknown
1	I have as much pep as I had in 2014.	U421
2	I am as happy now as I was younger.	U422
3	As I get older, things are better than I thought they would be.	U423

XXVI. PERCEIVED STRESS (for participants age 18 and older)

Table 17. Perceved Stress

1<br>Item No.	2<br>Statement<br>The questions in this table ask about your feelings and thoughts during the last month. In each case, you will be asked to indicate how often you felt or thought a certain way. Although some of the questions are similar, there are differences between them and you should treat each one as a separate question. The best approach is to answer each question fairly quickly. That is, don't try to count up the number of times you felt a particular way, but rather indicate the alternative that seems like a reasonable estimate. Please use 1-5 to describe if you never, almost never, sometimes, fairly often, very often had the feelings and thoughts.	3<br>1 Never<br>2 Almost never<br>3 Sometimes<br>4 Fairly often<br>5 Very often<br>9 Unknown
1	In the last month, how often have you been upset because of something that happened unexpectedly?	U551_
2	In the last month, how often have you felt that you were unable to control the important things in your life?	U552_
3	In the last month, how often have you felt nervous and stressed?	_ U553
4	In the last month, how often have you dealt successfully with irritating life hassles?	_ U554
5	In the last month, how often have you felt that you were effectively coping with important changes that were occurring in your life?	_ U555
6	In the last month, how often have you felt confident about your ability to handle your personal problems?	_ U556
7	In the last month, how often have you felt that things were going your way?	_ U557
8	In the last month, how often have you found that you could not cope with all the things that you had to do?	_ U558
9	In the last month, how often have you been able to control irritations in your life?	_ U559
10	In the last month, how often have you felt that you were on top of things?	_ U560
11	In the last month, how often have you been angered because of things that happened that were outside of your control?	_ U561
12	In the last month, how often have you found yourself thinking about things that you have to accomplish?	_ U562
13	In the last month, how often have you been able to control the way you spend your time?	_ U563
14	In the last month, how often have you felt difficulties were piling up so high that you could not overcome them?	_ U564

XXVII. DIET AND ACTIVITY KNOWLEDGE (for participants age 12 and older)

1. Do you know about the Chinese Pagoda or the Dietary Guidelines for Chinese Residents? _ U376
   - 0 No
   - 1 Yes

2. Do you proactively look for nutrition knowledge? _ U376a
   - 0 No (skip to Table 18.)
   - 1 Yes

3. Which is the most common way you use to look for nutrition knowledge? _ U376b
   - 1 Internet
   - 2 TV or Radio
   - 3 Books/Newspapers/Magazines
   - 4 Text or Apps through cell phones/smart phones
   - 5 From families/friends/classmates
   - 6 Lectures
   - 7 Other

*** Ask the respondent if he or she strongly agrees, somewhat agrees, somewhat disagrees or strongly disagrees with each statement in Item 4 and record the answers in Table 18.**

Table 18. Diet Knowledge

4<br>Statement<br><br>Do you strongly agree, somewhat agree, somewhat disagree or strongly disagree with this statement?<br>* Please note that the question is not asking about your actual habits.	5<br>1 strongly disagree<br>2 disagree<br>3 neutral<br>4 agree<br>5 strongly agree<br>9 unknown
Choosing a diet with a lot of fresh fruits and vegetables is good for one’s health.	_ U377
Eating a lot of sugar is good for one’s health.	_ U378
Eating a variety of foods is good for one’s health.	_ U379
Choosing a diet high in fat is good for one’s health.	_ U380
Choosing a diet with a lot of staple foods [rice and rice products and wheat and wheat products] is not good for one’s health.	_ U381
Consuming a lot of animal products daily (fish, poultry, eggs and lean meat) is good for one’s health.	_ U382
Reducing the amount of fatty meat and animal fat in the diet is good for one’s health.	_ U383
Consuming milk and dairy products is good for one’s health.	_ U384
Consuming beans and bean products is good for one’s health.	_ U385
Physical activities are good for one’s health.	_ U386
Sweaty sports or other intense physical activities are not good for one’s health.	_ U387
The heavier one’s body is, the healthier he or she is.	_ U388
Eating salty foods can cause hypertension.	_ U388a
Refined grains (rice and wheat flour) contain more vitamins and materials than unrefined grains.	_ U388b
Lard is healthier than vegetable oils.	_ U388c
Vegetables contain more starch than staple foods (rice or wheat flour).	_ U388d
Eggs and milk are the important sources of high-quality protein.	_ U388e

*** Ask the respondent if he or she likes very much, likes somewhat, dislikes somewhat, or dislikes very much each food in Item 6 and record the answers in Table 19.**

Table 19. Food Preferences

6<br>Food item<br><br>How much do you like this food: Like very much, like somewhat, dislike somewhat, or dislike very much?	7<br>1 dislike very much<br>2 dislike<br>3 neutral<br>4 like<br>5 like very much<br>9 does not eat this food
Fast food (KFC, pizza, hamburgers, etc.)	_ U389
Salty snack foods (potato chips, pretzels, French fries, etc.)	_ U390
Fruits	_ U391
Vegetables	_ U392
Soft drinks and sugared fruit drinks	_ U393

*** Ask the respondent if he or she likes very much, likes somewhat, dislikes somewhat, or dislikes very much each activity in Item 8 and record the answers in Table 20.**

Table 20. Activity Preferences

8<br>Activity type<br><br>How much do you like to participate in this activity: Like very much, like somewhat, dislike somewhat, or dislike very much?<br>* Please note we are asking if you participate in the activity, not just watch the activity or games on TV or as a spectator attending an event.	9<br><br>1 dislike very much<br>2 dislike<br>3 neutral<br>4 like<br>5 like very much<br>9 does not participate
Walking, Tai Chi	_ U394
Sports (ping pong, badminton, tennis, soccer, basketball, volleyball)	_ U395
Body building	_ U396
Watching TV	_ U397
Playing computer/video games, surfing the internet	_ U398
Reading	_ U399

*** Ask the respondent how important each of the priorities in Item 10 is in his or her life: The most important, very important, important, not very important, or not important at all and record the answers in Table 21a (for participants age 12 -17) or 21b (for participants age 18 and older).**

Table 21a. Priorities (for participants age 12 - 17)

10<br>Priority<br><br>How important is this priority in your life: The most important, very important, important, not very important, or not important at all?	11<br>1 not important at all<br>2 not very important<br>3 important<br>4 very important<br>5 the most important<br>9 unknown
Being praised by parents	_ U401
Being liked by friends	_ U402
Looking modern	_ U403
Getting good grades in school	_ U404

Table 21b. Priorities (for adults age 18 and older)

10<br>Priority<br><br>How important is this priority in your life: The most important, very important, important, not very important, or not important at all?	11<br>1 not important at all<br>2 not very important<br>3 important<br>4 very important<br>5 the most important<br>9 unknown
Having a good income	_ U405
Being physically active	_ U406
Eating a healthy diet	_ U407
Having my child be physically active	_ U408
Having my child eat a healthy diet	_ U409

XXVIII. FREQUCY OF CONSUMING SNACK FOODS AND PACKAGED FOODS (for participants age 6 and older)

1. Do you ever eat snack foods between two regular meals or in the evening after your main dinner? _ U511
   - 0 No (skip to Question 3)
   - 1 Yes
   - 9 Unknown (skip to Question 3)
2. How often do you usually have snack foods? _ U512
   - 1 Almost every day
   - 2 More than 4 times a week but not everyday
   - 3 3 – 4 times a week
   - 4 1 -2 times a week
   - 5 1 – 3 times a month
   - 6 Less than once a month
   - 9 Unknown
3. Do you ever eat food that is packed in a box, bag or bottle with a label and name and is either Packaged pre-cooked foods, Packaged cooked foods, Packaged non-cooked foods, Packaged processed foods (added flavor, added items with and without nutritive value), or Packaged non-processed foods? _ U513
   - 0 No (skip to the next section)
   - 1 Yes
   - 9 Unknown (skip to the next section)
4. How often do you usually eat packaged foods? _ U514
   - 1 Almost every day
   - 2 More than 4 times a week but not everyday
   - 3 3 – 4 times a week
   - 4 1 -2 times a week
   - 5 1 – 3 times a month
   - 6 Less than once a month
   - 9 Unknown
5. Do you usually read the Nutrition Facts like below when you buy packaged foods? _ U515

**Nutrition Facts**	Amount / Serving	% Daily Value*	Amount / Serving	% Daily Value*
Serving Size 2 turtles (38g)	**Total Fat** 18g	**28%**	**Total Carbohydrate** 16g	**5%**
Servings Per Container 6	Saturated Fat 4g	**20%**	Dietary Fiber 2g	**8%**
**Calories** 220	Trans Fat 0g		Sugars 11g	
Calories from Fat 160	**Cholesterol** 5mg	**2%**	**Protein** 2g	
	**Sodium** 20mg	**1%**		
*Percent Daily Values are based on a 2,000 calorie diet.	Vitamin A 2% • Vitamin C 0%		Calcium 4% • Iron 4%	

   - 1 Never
   - 2 Seldom (skip to question 7)
   - 3 Sometimes (skip to question 7)
   - 4 Often (skip to question 7)
   - 5 Always (skip to question 7)
6. What is the major reason that you do not read the Nutrition Facts? _ U516
   - 1 Seldom buy packaged foods
   - 2 Have limited time on shopping for foods
   - 3 Do not realize that there are Nutrition Facts on the packages
   - 4 Do not understand the meaning of Nutrition Facts
   - 5 Nutrition Facts are less important than price, tastes, or other factors
   - 6 Already know Nutrition Facts among different foods and do no need to read
   - 7 Other reasons

7. **How often do you usually pay attention to the following components when you read the Nutrition Facts?**
***Use 1-5 to describe your attention from the least frequency to the most frequency and record in table 22.**

Table 22. Priorities

7<br>Attention to each nutrient component<br><br>How often do you usually pay attention to the following components when you read the Nutrition Facts, never, seldom, sometimes, often, or always?	8<br>1 Never<br>2 Seldom<br>3 Sometimes<br>4 Often<br>5 Always<br>9 unknown
Energy	U517a
Protein	U517b
Fat	U517c
Carbohydrates	U517d
Sodium	U517e

XXIX. MARRIAGE HISTORY (for all women under age 52 who are married, widowed, or divorced)

1. What is your current marital status? _ S1
   2 married
   3 divorced (skip to Question 4)
   4 widowed (skip to Question 4)

2. In what year and month were you married? (current marriage) _ _ _ _ year _ _ month S2
   ____year ____month
   * Record western calendar, if possible.

3. Does your husband ordinarily live at home? _ S3
   0 no
   1 yes
   *** Skip to Question 6**

4. In what year and month were you and your most recent husband married?____year ____month _ _ _ _ year _ _ month S4
   * Record western calendar, if possible.

5. In what year and month were you most recently widowed or divorced? ____year ____month _ _ _ _ year _ _ month S5
   * Record western calendar, if possible.

6. Altogether, how many times have you been married? ____times. _ S35

XXX. INTER-GENERATIONAL LINKAGES TO PARENTS(for all women under age 52 who are married, widowed, or divorced)

*** Ask Questions 2-7 about inter-generational linkages and record the answers in Table 23.**

Table 23. Inter-generational Linkages to Parents

1	2<br>Is s/he still alive?<br><br>0. No(skip_ to next relative)<br>1. Yes	3<br>Where does s/he live?<br>1. Same household<br>2. Next door or adjacent to household<br>3. Same neighborhood/ village<br>4. Outside neighborhood, but same city or county<br>5. Other city or county<br>9. Unknown	4<br>Is s/he over age 50?<br><br>0. No(skip to next relative)<br>1. Yes	5<br>Does s/he need to be taken care of (refers to the need for other people's help in daily life and shopping)?<br>0. No<br>1. Yes	6<br>During the past week, did you help her/him with her/him daily life and shopping?<br>0. No(skip to next relative)<br>1. Yes	7<br>During the past week, how much time did you spend taking care of her/him?
Mother	S6	S7	S10a	S11	S11a	S12
Father	S13	S14	S17a	S18	S18a	S19
Mother-in-law	S20	S21	S24a	S25	S25a	S26
Father-in-law	S27	S28	S31a	S32	S32a	S33

XXXI. SIBLINGS/RELATIVES(for all women under age 52 who are married, widowed, or divorced)

1. Do you have any brothers? S215
   - 0 no (skip to Question 3)
   - 1 Yes
2. How many brothers do you have? S216
3. Do you have any sisters? S217
   - 0 no (skip to Question 5)
   - 1 Yes
4. How many sisters do you have? S218

*** Ask Questions 5-8 for currently married women only.**

5. Does your husband have any brothers? S219
   - 0 no (skip to Question 7)
   - 1 Yes
6. How many brothers does your husband have? S220
7. Does your husband have any sisters? S221
   - 0 no (skip to the next section)
   - 1 Yes
8. How many sisters does your husband have? S222

XXXII. PREGNANCY HISTORY(for all women under age 52 who are married, widowed, or divorced)

1. Are you currently pregnant? S59
   - 0 no (skip to Question 3)
   - 1 Yes
   - 9 unknown (skip to Question 3)

2. For how many months have you been pregnant? U57
   * If "unknown," record -9.
   *** Skip to Question 7**

3. Are you using any contraceptive methods? S65
   - 0 no
   - 1 Yes(skip to Question 5)

4. What is the reason that you do not use contraceptive methods? S71

01	want to have a child	07	inconvenient to use
02	one part of the couple is sterile	08	infrequent sex
03	husband or relatives disapprove	09	husband not living at home
04	health reason	10	husband deceased or divorced
05	unacceptable or inaccessible	11	fatalistic attitude
06	cost too much	12	other (specify: __________)

5. From January 2011 to the present, how many times have you been pregnant, including the current pregnancy if currently pregnant? S109
   *** If "none," skip to the next section. *For new participants, ask their pregnancies to date.**

Table 24. Pregnancy History: January 2011 to Present

*** Ask Questions 6-13 about each pregnancy since January 2011 (since the very first one for new participants) that has ended already (excluding the current one if currently pregnant) and record the answers in Table 24.**

* Begin with the most recent pregnancy (excluding the current one) and work backward, recording up to 5 pregnancies.

* Record western calendar, wherever possible.

* If the current pregnancy is the only pregnancy since January 2011, skip to the next section**.**

6<br>Pregnancy number<br>**pregnum**	7<br>When did this pregnancy end? (year, month, day)<br>**S113a**	8<br>How did this pregnancy end?<br>1 natural abortion<br>2 induced abortion<br>3 stillborn fetus (<7 mo)<br>4 stillbirth (>7 mo)<br>5 live birth (ask Q11-15)<br>* If not a live birth (code 1-4), ask about next pregnancy.<br>**S114**	9<br>What was this child's sex?<br>1 male<br>2 female<br>**S114a**	10<br>Is this child still alive?<br>0 no (skip to Question 14)<br>1 yes<br>**S114c**	11<br>What is this child's name?<br>*Record child's line number<br>**line_c**	12<br>Did you ever breastfeed this child?<br>0 no<br>1 yes, now<br>2 yes, no longer<br>**S116**	13<br>How long did you breastfeed this child? (months)<br>**S117**
1	_ _ _ _ _	_	_	_	_ _ _	_	_ _
2	_ _ _ _ _ _ _ _ _	_	_	_	_ _ _	_	_ _
3	_ _ _ _ _ _ _ _ _	_	_	_	_ _ _	_	_ _
4	_ _ _ _ _ _ _ _ _	_	_	_	_ _ _	_	_ _
5	_ _ _ _ _ _ _ _ _	_	_	_	_ _ _	_	_ _

*** Ask Questions14-15 about the most recent pregnancy (excluding the current one)**.

14. Did you have prenatal care during this pregnancy? _ S86
    - 0 no (skip to the next section)
    - 1 Yes

15. How many prenatal examinations did you have altogether? _ _ S88

XXXIII. BIRTH HISTORY (for all women under age 52 who are married, widowed, or divorced, and who have given birth to a child)

*** We have asked about pregnancies and births since January 2011(since the very first one for new participants). Now we will ask about all the children you have ever given birth to. Please answer all questions for all children, including those who died and those born recently.**

*** Ask Questions 2-10 about every child the woman has given birth to (including those who died and those born since January 2011), and record the answers in Table 25.**

* Begin with the first birth and work forward to the most recent birth.

* Record western calendar, wherever possible.

Table 25. Birth History

1<br>Birth order<br>**S48**	2<br>When was this child born? (year, month, day)<br>**AA3**	3<br>According to which calendar?<br>1 western<br>2 lunar<br>**AA4**	4<br>What was this child's sex?<br>1 male<br>2 female<br>**gender**	5<br>Is this child living with you now?<br>0 no (skip to Q7)<br>1 yes<br>**S52**	6<br>What is this child's name?<br>* Record child's line number.<br>* Ask about next child.<br>**line_c**	7<br>Is this child living elsewhere?<br>0 no<br>1 yes (skip to Q10)<br>**S54**	8<br>When did this child die? (year, month)<br>**S56**	9<br>Was this child living in your household when he or she died?<br>0 no<br>1 yes<br>**S57**	10<br>How long did this child live in your household? (years, months)<br>**S58**
1	_ _ _ _ _ _ _ _	_	_	_	_ _ _	_	_ _ _ _ _ _	_	_ _ years _ _ months
2	_ _ _ _ _ _ _ _	_	_	_	_ _ _	_	_ _ _ _ _ _	_	_ _ years _ _ months
3	_ _ _ _ _ _ _ _	_	_	_	_ _ _	_	_ _ _ _ _ _	_	_ _ years _ _ months
4	_ _ _ _ _ _ _ _	_	_	_	_ _ _	_	_ _ _ _ _ _	_	_ _ years _ _ months
5	_ _ _ _ _ _ _ _	_	_	_	_ _ _	_	_ _ _ _ _ _	_	_ _ years _ _ months
6	_ _ _ _ _ _ _ _	_	_	_	_ _ _	_	_ _ _ _ _ _	_	_ _ years _ _ months
7	_ _ _ _ _ _ _ _	_	_	_	_ _ _	_	_ _ _ _ _ _	_	_ _ years _ _ months
8	_ _ _ _ _ _ _ _	_	_	_	_ _ _	_	_ _ _ _ _ _	_	_ _ years _ _ months
9	_ _ _ _ _ _ _ _	_	_	_	_ _ _	_	_ _ _ _ _ _	_	_ _ years _ _ months
10	_ _ _ _ _ _ _ _	_	_	_	_ _ _	_	_ _ _ _ _ _	_	_ _ years _ _ months

XXXIV. EATING DISORDERS (for girls and women age 12 to 49 years old)

1. Do you make yourself Sick because you feel uncomfortably full? _ Z1
   - 0 No
   - 1 Yes
   - 9 Unknown

2. Do you worry that you have lost Control over how much you eat? _ Z2
   - 0 No
   - 1 Yes
   - 9 Unknown

3. Have you recently lost more than 6.35 kg (12.7 jin) in a 3-month period? _ Z3
   - 0 No
   - 1 Yes
   - 9 Unknown

4. Do you believe yourself to be Fat when others say you are too thin? _ Z4
   - 0 No
   - 1 Yes
   - 9 Unknown

5. Would you say that Food dominates your life? _ Z5
   - 0 No
   - 1 Yes
   - 9 Unknown

*** Ask the respondent about her dietary behaviors in past 4 weeks (28 days) and record in Table 26.**

Table 26. Dietary Behaviors in past 4 Weeks

6<br>**Dietary Behaviors**<br><br>The total days when you have the following dietary behaviors<br><br>* We are asking about if you had the following behaviors, whether or not successful.	7<br><br>0 no<br>1 1-5 days<br>2 6-12 days<br>3 13-15 days<br>4 16-22 days<br>5 23-27 days<br>9 daily
Have you been deliberately trying to limit the amount of food you eat to influence your shape or weight?	_ Z6
Have you gone for long periods of time (8 hours or more) without eating anything in order to influence your shape or weight?	_ Z7
Have you tried to avoid eating any foods which you like in order to influence your shape or weight?	_ Z8
Have you tried to follow definite rules regarding your eating in order to influence your shape or weight; for example, a calorie limit, a set amount of food, or rules about what or when you should eat?	_ Z9
Have you wanted your stomach to be empty?	_ Z10
Have you felt fat?	_ Z11
Have you had a strong desire to lose weight?	_ Z12

XXXVI. FIRST MENSTRUATION (for girls age 8 - 17)

1. Have you ever menstruated? _ U20
   - 0 no (skip to the next section)
   - 1 yes
   - 9 unknown (skip to the next section)

2. At what age did you first menstruate? (years) ________ _ _ U21
   * If "unknown," record -9.

XXXVII. Boy Maturation (for boys age 10-17)

To interviewer: Please write down boy's household ID and Line number, then ask boys age 10 – 17 to fill in the form and return to interviewer

3. Has your voice changed in the past few years? _ BY29
   - 0 No (skip to question 5)
   - 1 Yes
   - 9 Unknown (skip to question 5)

4. How different is your voice now than when you were 10 years old? _ BY30
   - 0 Has changed a bit
   - 1 Has changed a lot
   - 9 Unknown

5. How do you compare your body with that of other boys your age? _ BY31
   - 1 Younger
   - 2 The same as other boys
   - 3 More mature
   - 9 Unknown

6. Have you started shaving the hair on your face? _BY32
   - 0 No
   - 1 Yes
   - 9 Unknown

7. How thick is the hair under your arms? _ BY33
   - 0 No hair
   - 1 Just a little
   - 2 Very thick

8. Please look at the drawings and read what is written underneath each box. Choose which drawing matches the thickness of your hair and mark the corresponding box with a circle. Remember to focus on the pubic hair and not the penis. _ BY34

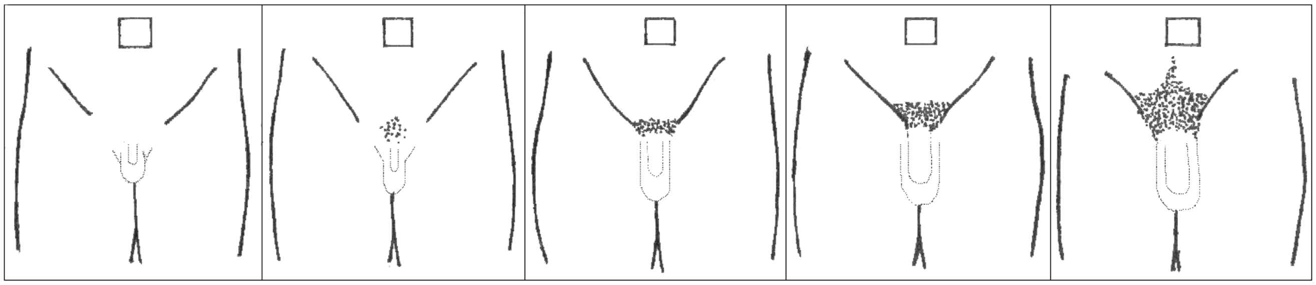

1	2	3	4	5
There is no hair yet.	There are a few which are fine, long and not so dark. Most of the hair is around the base of the penis and is either curly or straight.	The hair is darker and curlier. It has spread out and thinly covers a bigger area.	The hair is really dark as that of an older man but still not a lot. The hair has not spread out to the legs.	The hair has spread out to the legs. There is more hair as that of an older man.
